# Chemoproteomic target deconvolution reveals Histone Deacetylases as targets of (*R*)-lipoic acid

**DOI:** 10.1038/s41467-023-39151-8

**Published:** 2023-06-15

**Authors:** Severin Lechner, Raphael R. Steimbach, Longlong Wang, Marshall L. Deline, Yun-Chien Chang, Tobias Fromme, Martin Klingenspor, Patrick Matthias, Aubry K. Miller, Guillaume Médard, Bernhard Kuster

**Affiliations:** 1 Chair of Proteomics and Bioanalytics, TUM School of Life Sciences, Technical University of Munich, Freising, Germany; 2 Cancer Drug Development, German Cancer Research Center (DKFZ), Heidelberg, Germany; 3 Biosciences Faculty, Heidelberg University, Heidelberg, Germany; 4 Friedrich Miescher Institute for Biomedical Research, 4058 Basel, Switzerland; 5 Faculty of Sciences, University of Basel, 4031 Basel, Switzerland; 6 Chair of Molecular Nutritional Medicine, TUM School of Life Sciences, Technical University of Munich, Freising, Germany; 7 EKFZ - Else Kröner Fresenius Center for Nutritional Medicine, Technical University of Munich, Freising, Germany; 8 ZIEL Institute for Food & Health, Technical University of Munich, Freising, Germany; 9 German Cancer Consortium (DKTK), Heidelberg, Germany; 10 Bavarian Center for Biomolecular Mass Spectrometry (BayBioMS), Technical University of Munich, Freising, Germany

**Keywords:** Small molecules, Target identification, Proteomics, Histone post-translational modifications

## Abstract

Lipoic acid is an essential enzyme cofactor in central metabolic pathways. Due to its claimed antioxidant properties, racemic (*R*/*S*)-lipoic acid is used as a food supplement but is also investigated as a pharmaceutical in over 180 clinical trials covering a broad range of diseases. Moreover, (*R*/*S*)-lipoic acid is an approved drug for the treatment of diabetic neuropathy. However, its mechanism of action remains elusive. Here, we performed chemoproteomics-aided target deconvolution of lipoic acid and its active close analog lipoamide. We find that histone deacetylases HDAC1, HDAC2, HDAC3, HDAC6, HDAC8, and HDAC10 are molecular targets of the reduced form of lipoic acid and lipoamide. Importantly, only the naturally occurring (*R*)-enantiomer inhibits HDACs at physiologically relevant concentrations and leads to hyperacetylation of HDAC substrates. The inhibition of HDACs by (*R*)-lipoic acid and lipoamide explain why both compounds prevent stress granule formation in cells and may also provide a molecular rationale for many other phenotypic effects elicited by lipoic acid.

## Abstract (web summary)

Lipoic acid is a food supplement and approved neuropathy drug but lacks known molecular targets. Here, Lechner *et al*. employ chemoproteomic target deconvolution to demonstrate that (*R*)- but not (*S*)-lipoic acid stereoselectively inhibits histone deacetylases at physiologically achievable doses.

## Introduction

The disulfide-containing fatty acid lipoic acid (LA) is an endogenously produced molecule and is essential for aerobic metabolism both in prokaryotes and eukaryotes^1^. When attached via an amide bond to a lysine side chain (lipoylation), (*R*)-LA acts as a cofactor for several enzymes including the pyruvate dehydrogenase complex at the intersection between glycolysis and the citric acid cycle^1^. Lipoic acid can be synthesized in cells but is also taken up by cells from exogenous sources. The racemic mixture of LA enantiomers ((*R*/*S*)-LA) is used as a food supplement and as a therapeutic drug, purportedly because of its property as an antioxidant^2^. This is attributed to the fact that, following cellular uptake, the disulfide bond in LA is readily reduced^3^ and the thiols may chelate metal ions or scavenge reactive oxygen or nitrogen species. At the time of writing, LA was subject to 187 clinical trials (22 phases 4 trials; clinicaltrials.gov) covering, for instance, endocrine, neurological, and autoimmune diseases such as diabetes mellitus, peripheral nervous system disorders, or multiple sclerosis, respectively^4^. Lipoic acid has been proven efficacious in diabetic neuropathy^5^, which affects approximately 16% of diabetes patients^6^ and, as such, is frequently prescribed for treating diabetic neuropathy and neuropathic pain^7–9^. Lipoic acid is well tolerated at clinically relevant doses ranging from 600 to 2400 mg/day (both orally and intravenously) and reaches peak plasma concentrations of 100–500 µM^10^. Despite its widespread use, the mechanism of action (MoA) of LA remains unclear. Much attention has been placed on its redox properties and ability to scavenge reactive oxygen species (ROS). Intriguingly, lipoic acid and the closely related amide lipoamide (LM) were recently discovered as hits in an in vitro screen for molecules that disrupt stress granules in amyotrophic lateral sclerosis (ALS) model systems^11^. However, the targets underlying the observed phenotype remain elusive.

Here, we applied a chemoproteomic approach to identify direct protein targets of LA and LM. This revealed that Zn^2+^-dependent histone deacetylases (HDACs) are protein targets of racemic (*R*/*S*)-LM as well as of (*R*)-LA, but not (*S*)-LA. (*R*)-LA and (*R*/*S*)-LM bind HDACs with low two-digit µM affinities and inhibit their enzymatic activity resulting in increased acetylation of HDAC substrates. The inhibition of HDACs also affects the hyperacetylation of the stress granule-forming protein DDX3X. LM and (*R*)-LA but not the inactive (*S*)-enantiomer prevented the formation of stress granules in A549 cells, suggesting that HDAC inhibition is the cellular MoA underlying this cellular phenotype.

## Results

### HDACs are targets of lipoic acid and LM

**Fig. 1:**
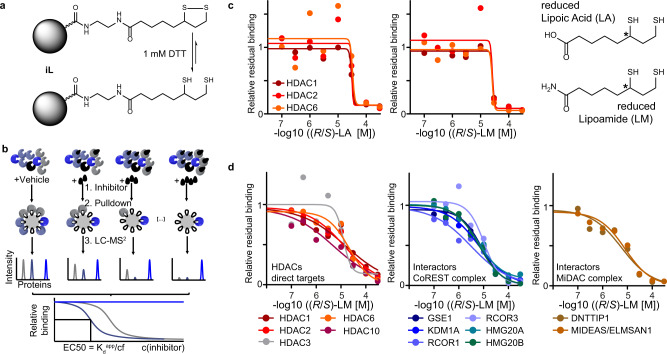
Chemoproteomics identifies HDACs as targets of lipoic acid and lipoamide. **a** An affinity matrix iL was synthesized by immobilizing racemic (*R*/*S*)-lipoic acid to sepharose beads. The resulting affinity matrix resembles lipoamide and is reduced to dihydrolipoamide under assay conditions (1 mM DTT). **b** Schematic representation of the competition pulldown assay used in this study. Lysate containing correctly folded proteins interacting with endogenous cofactors or macromolecular binding partners is incubated with the affinity matrix to pull down target proteins. In a competition experiment, the lysate is first incubated with different doses of the free drug of interest (black droplet symbol) before pull down. LC–MS/MS is used to quantify target proteins. The intensities are plotted against the drug concentration to yield dose-response curves, from which binding EC50s and *K*_d_^app^ can be derived (cf=correction factor; see “Methods”). **c** Dose–response curves for lipoic acid and lipoamide using a lysate of SW620 cancer cells. Structures of drugs are shown in the reduced form and the chiral center is indicated by an asterisk. **d** Dose–response curves for (*R*/*S*)-LM using a lysate of MV4–11 cancer cells showing HDACs and HDAC complex partners of the CoREST (blue) and MiDAC (brown) complexes. Source data are provided as a Source Data file.

To identify proteins directly bound by lipoic acid, we employed a chemoproteomic competition assay^12^. Briefly, (*R*/*S*)-LA was immobilized on sepharose beads via an amidation reaction to form an affinity matrix (abbreviated as iL; Fig. 1a). This affinity matrix can be incubated with cell lysate to pull down potential LA or LM target proteins and identify these targets via bottom-up proteomics. In a competition assay, free LA or LM were incubated with lysate at different concentrations before pulling down and quantifying target proteins. This pre-incubation leads to the dose-dependent reduction of pulled-down target proteins and allows to derive EC50 (effective concentration to reduce affinity matrix binding by 50%) values as well as apparent dissociation constants (*K*_d_^app^, expressed as p*K*_d_^app^ = −log_10_ *K*_d_^app^)^13^ (Fig. 1b, see “Methods” for details). Pulldowns using a lysate of the colorectal cancer cell line SW620 showed clear dose-dependent competition of HDAC1, HDAC2 (including co-competed members of the HDAC1/2 containing CoREST complex), and HDAC6 for both (*R*/*S*)-LA and (*R*/*S*)-LM with affinities in the range of 3–33 µM (Fig. 1c, Supplementary Fig. 1a, Supplementary Data 1). The lysate buffer used in this assay contains 1 mM DTT to mimic the intracellular reductive milieu and to reduce lipoic acid and LM. The same experiment performed using the leukemia cell line MV4-11 validated HDAC1 (*K*_d_^app^ = 16 µM), HDAC2 (*K*_d_^app^ = 14 µM), and HDAC6 (*K*_d_^app^ = 21 µM) and identified HDAC3 (*K*_d_^app^ = 13 µM) and HDAC10 (*K*_d_^app^ = 5 µM) as additional targets of (*R*/*S*)-LM (Fig. 1d). Additional competition assays performed in lung adenocarcinoma cell A549 lysate also identified HDAC1, HDAC2, and HDAC6 as targets (Supplementary Fig. 1b). Amongst the three cell lines tested, HDACs were the only confidently identified targets across the 1500–3000 proteins quantified in these assays. Several HDAC complex members were co-competed, suggesting that LA and LM also engage the class I HDACs as part of gene regulatory complexes (Fig. 1d, Supplementary Fig. 1a–d). We validated the results by analogous pulldown competition assays using two other affinity matrices prepared via the immobilization of either the HDAC inhibitor Quisinostat (iQ) and an analog of the class IIa HDAC inhibitor Bürli’s 31^14^ (iC)^12^. We have previously shown that these affinity matrices specifically bind to the active sites of HDACs and have used them for HDACi selectivity profiling^12^. Indeed, binding of (*R*/*S*)-LA and (*R*/*S*)-LM to HDAC1, HDAC2, HDAC3, HDAC6, and, to a lesser extent, HDAC8 was confirmed in these assays (Supplementary Fig. 1c, d). No binding to class IIa HDACs or the recently discovered common HDAC inhibitor off-target MBLAC2 was observed (Supplementary Fig. 1e)^12^. Interestingly, the competition assay data indicated that (*R*/*S*)-LA does not bind HDAC10, while (*R*/*S*)-LM binds HDAC10 with an even higher affinity than other HDACs. This observation might reflect electrostatic repulsion between the HDAC10 active site gate-keeper glutamic acid residues^15,16^ and the terminal carboxylic acid of LA.

### The reduced forms of (*R*)-lipoic acid and racemic LM inhibit HDACs

**Fig. 2:**
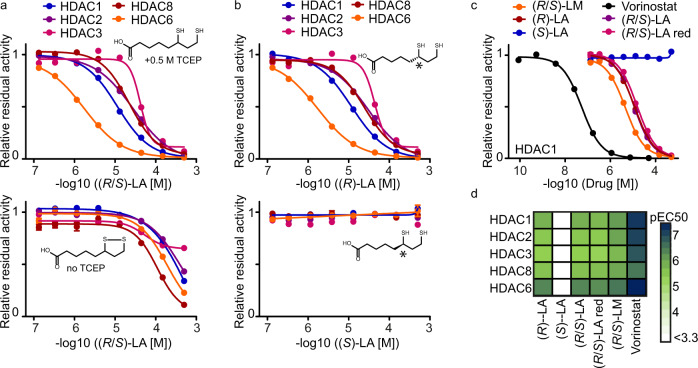
HDAC activity assays confirm the inhibitory effects of the reduced forms of (*R*)-lipoic acid, (*R*/*S*)-lipoic acid, and (*R*/*S*)-lipoamide. **a** Influence of the reducing agent TCEP (0.5 M) on (*R*/*S*)-LA mediated HDAC enzymatic activity via reduction and ring opening of the drug (for (*R*/*S*)-LM see Supplementary Fig. 2b) (*n* = 3 technical replicates, data are represented as mean value ± SEM). **b** HDAC inhibitory effect of the (*R*)-enantiomer of lipoic acid compared to the (*S*)-enantiomer (*n* = 3 technical replicates, data are represented as mean value ± SEM). **c** Exemplary dose-response profiles of all compounds tested for HDAC1 inhibition in the presence of 0.5 M TCEP ((*R*/*S*)-LA red = (*R*/*S*)-dihydrolipoic acid). *n* = 3 technical replicates. **d** Summary of EC50 values derived from dose-dependent HDAC inhibition curves. Source data are provided as a Source Data file.

To validate the inhibition of HDACs by LA and LM and elaborate on the structure-activity relationship (SAR) of enantiomers as well as oxidized versus reduced forms of the molecules, we performed enzyme activity assays for recombinant HDAC1,2,3,6,8 as well as FRET-based binding assay for recombinant HDAC10. We included the clinically approved HDAC inhibitor Vorinostat (SAHA) as a positive control. To measure the potential dependence of HDAC inhibition on the redox state of the molecules, experiments were performed in the presence or absence of TCEP, a reducing agent with no effect on HDAC activity and HDAC inhibitor binding under the assay conditions (Supplementary Fig. 2a). Reduced (*R*/*S*)-LA and (*R*/*S*)-LM inhibit HDAC1,2,3,6,8 (EC50 = 1–44 µM) while the oxidized form of (*R*/*S*)-LA showed diminished activity. No activity at all was detected for oxidized (*R*/*S*)-LM (Fig. 2a, Supplementary Fig. 2b, c). Performing the same experiment using reduced racemic dihydrolipoic acid yielded affinity values comparable to racemic lipoic acid in the presence of TCEP, suggesting complete reduction and dithiolane ring opening under the assay conditions (Supplementary Fig. 2d). As observed in the chemoproteomic experiments, potent HDAC10 binding of (*R*/*S*)-LM could be confirmed. While (*R*/*S*)-LM affinity for HDAC10 was only 5-fold lower than that of Vorinostat, (*R*/*S*)-LA showed almost 200-fold lower affinity to HDAC10 compared to Vorinostat (Supplementary Fig. 2e). The (*S*)-enantiomer of lipoic acid had no activity against any HDAC at concentrations of up to 500 µM (Fig. 2b, c). This makes (*S*)-LA an ideal negative control for the assessment of which of the phenotypic effects elicited by lipoic acid are related to HDAC inhibition and which are related to other physicochemical properties of the molecule such as metal ion chelation or ROS scavenging.

In summary, the above data indicate that reduced (*R*)-LA, as well as (*R*/*S*)-LM feature an HDAC selectivity profile similar (albeit less potent) compared to that of clinical drugs such as Vorinostat and Ricolinostat (Fig. 2d, Supplementary Fig. 2f). All these molecules inhibit HDAC1–3 with similar affinity and HDAC6 with 5- to 15-fold higher relative potency. Given that the cytosol is a disulfide-reducing environment, and lipoic acid is readily reduced once inside the cell^3^, both lipoic acid and LM, most probably, exist in cells in their reduced and, therefore, HDAC-inhibiting forms.

### (*R*)-lipoic acid engages HDACs in cells and induces HDAC substrate hyperacetylation

**Fig. 3:**
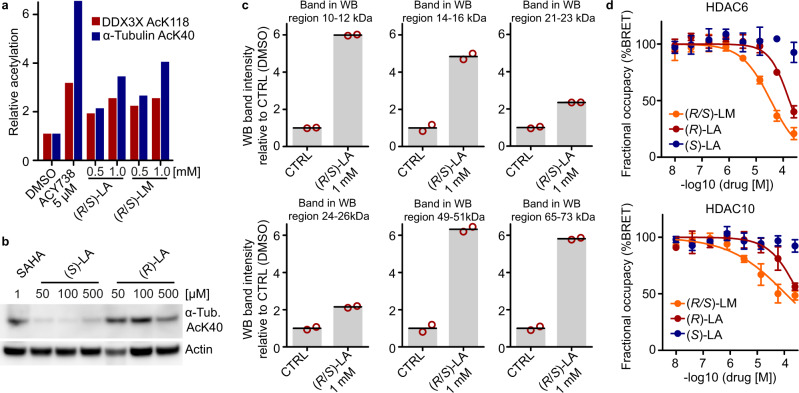
(*R*/*S*)-lipoic acid and (*R*/*S*)-lipoamide lead to hyperacetylation of HDAC substrates in cells. **a** Western blot analysis of acetylation levels of HDAC6 substrates following 12 h treatment of HEK293T cells with (*R*/*S*)-LA, (*R*/*S*)-LA, and the HDAC6 inhibitor ACY738 (see also Supplementary Fig. 3a). **b** Western blot for α-Tubulin AcK40 acetylation levels after 12 h treatment of A549 cells with SAHA (Vorinostat), (R)-LA, and (S)-LA. **c** Western blot analysis for global lysine acetylation levels of HeLa S3 cells treated with (*R*/*S*)-LA (16 h; *n* = 2 independent biological experiments, error bars represent standard deviation; see also Supplementary Fig. 3c). The histograms show hyperacetylation of proteins in the size range of established HDAC substrates, such as Histones (11–16 kDa), Peroxiredoxin (22 kDa)^20^, α-Tubulin (50 kDa), and others. **d** HDAC6 and HDAC10 nano-BRET assays demonstrating in-cellulo target engagement in HEK293T cells (*n* = 3 independent experiments, data are represented as mean value ± SD; curve fitted with a variable slope; bottom constrained to 0 and top constrained to 100). Source data are provided as a Source Data file.

To verify that (*R*)-LA and (*R*/*S*)-LM inhibit HDACs in cells, we treated a set of cell lines (HEK293T, A549, HeLa S3) with racemic or enantiomerically pure lipoic acid, racemic LM, the HDAC6/MBLAC2 inhibitor Tubacin, the potent HDAC6 selective inhibitor ACY-738^12^, and the unselective HDAC1,2,3,6 inhibitor Vorinostat and probed for HDAC substrate hyperacetylation. Western blot analysis confirmed that (*R*/*S*)-LA and (*R*/*S*)-LM dose-dependently increased acetylation levels of the well-established HDAC6 substrate α-Tubulin AcK40 (Supplementary Fig. 3a) and showed a substantial increase of acetylation at concentrations as low as 50 µM after 7 h of drug incubation. In another experiment, HEK293T cells were probed for acetylation of the stress granule protein DDX3X K118, another well-established HDAC6 substrate site^17^. Again lipoic acid and LM both dose-dependently increased acetylation levels of α-Tubulin AcK40 (3.4-fold with 1 mM (*R*/*S*)-LA; 4.0-fold with 1 mM (*R*/*S*)-LM) and DDX3X AcK118 (2.4-fold at 1 mM (*R*/*S*)-LA and (*R*/*S*)-LM). The extent of DDX3X hyperacetylation induced by 1 mM (*R*/*S*)-LA or (*R*/*S*)-LM was in the same range as for ACY-738 (3.0-fold at 5 µM) (Fig. 3a, Supplementary Fig. 3b) and the HDAC6/10 inhibitor Tubastatin A (ref. ^17^, 2.6-fold at 10 µM). Importantly, in contrast to HDAC-inhibiting (*R*)-lipoic acid and Vorinostat, (*S*)-lipoic acid did not lead to a substantial increase of α-Tubulin AcK40 or Histone H4 AcK5/8/12/16 in HEK293T cells or A549 lung adenocarcinoma cells at concentrations of up to 500 µM (Fig. 3b, Supplementary Fig. 3c). HDAC substrate hyperacetylation is commonly used to demonstrate in-cellulo inhibition of HDACs and our data, therefore, provides evidence for HDAC-inhibitory activity of (*R*)-lipoic acid and (*R*/*S*)-LM in cells. Of note, (*R*/*S*)-lipoic acid has previously been shown to increase α-Tubulin acetylation but direct HDAC6 inhibition was not proposed as the underlying mode of action^18^. Given that LA and LM inhibit several HDACs (nuclear HDAC1, HDAC2, HDAC3, HDAC8, and cytosolic HDAC6) that collectively have hundreds of substrates^19^, we reasoned that LA and LM may increase acetylation levels of many cellular proteins. Indeed, western blot analysis for global acetylation levels in (*R*/*S*)-LA treated HeLa S3 cells showed elevated levels of acetylation of proteins in the size range of histones (11–16 kDa), α-Tubulin (50 kDa) and others (Fig. 3c, Supplementary Fig. 3c). Similarly, time-dependent treatment of HeLa S3 cells with (*R*/*S*)-LA clearly showed upregulation of acetylation on a broad range of proteins within 1 h and peaking between 3–8 h (Supplementary Fig. 3d, e). To show that hyperacetylation is a direct result of HDAC inhibition, we performed nanoBRET-based intracellular target engagement assays using (*R*)-LA, (*S*)-LA, (*R*/*S*)-LM, and Vorinostat against HDAC6 and HDAC10 in HeLa cells. Indeed, (*R*)-LA as well as (*R*/*S*)-LM showed dose-dependent intracellular binding to HDAC6 and HDAC10, while (*S*)-LA was inactive. HDAC6 was half maximally inhibited at 35 µM (*R*/*S*)-LM and 170 µM (*R*)-LA. These values agree well with the observed HDAC substrate hyperacetylation using 2–3 digit µM doses of the molecules and are below the clinically observed maximal peak plasma concentration of lipoic acid. Together, these results suggest that lipoic acid and LM engage and inhibit HDACs in cells.

### (*R*)-lipoic acid and (*R*/*S*)-LM inhibit stress granule formation in cells

**Fig. 4:**
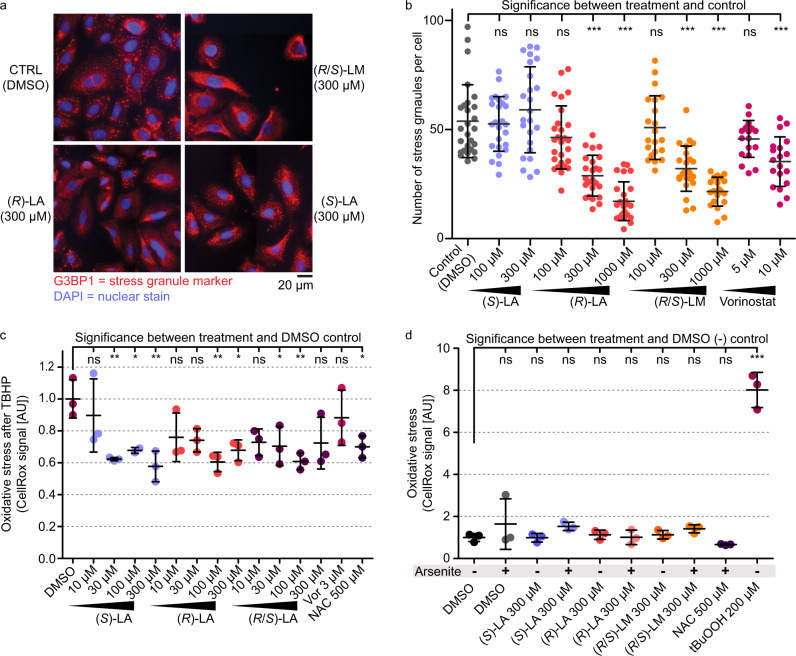
Lipoic acid and lipoamide reduce stress granule formation in cells. **a** Immunofluorescence detection of the stress granule marker G3BP1 in A549 cancer cells. Stress granules appear as red foci in the DMSO control and cells treated with (HDAC-inactive) (*S*)-LA. The reduction of defined stress granules in response to (*R*)-LA and (*R*/*S*)-LA is apparent from the blurred red areas. **b** Quantification of the number of stress granules per cell. Each treatment was performed in *n* = 3 independent biological experiments and between 140 and 150 cells were submitted to stress granule counting. **c** Levels of oxidative stress induced by 2 h treatment with 200 µM Tertbutylhydroperoxide (BuOOH) after 1 h pre-treatment with drugs (Vor Vorinostat, NAC N-acetylcysteine) in A549 cells. Oxidative stress levels were assessed using the CellRox assay. Every data point corresponds to one biological replicate and is the mean CellRox intensity from 9 to 10 pictures capturing 60–180 cells in total (*n* = 2 biologically independent samples for 100 µM (*S*)-LA, *n* = 3 biologically independent samples for all other treatments, AU arbitrary units). **d** Levels of oxidative stress in A549 cells after 1.5 h drug pre-treatment, optionally followed by a 30 min arsenite (1 mM) pulse. Oxidative stress levels were assessed using the CellRox assay. Every data point corresponds to one biological replicate and equals the mean CellRox intensity from 10 to 15 pictures capturing 60–180 cells in total (*n* = 3 biologically independent samples for each drug dose, AU arbitrary units). **b**–**d** Statistical significance was calculated between the control and drug pre-treatments by one-way ANOVA following the Dunnett test for multiple comparisons using the GraphPad Prism software. Data are presented as means ± SD. ns not significant, ***P-value ≤ 0.001, **P-value ≤ 0.01, *P-value ≤ 0.05 in one-way ANOVA after Dunnett’s multiple comparison test). Source data are provided as a Source Data file.

The acetylation status of proteins can affect their macromolecular associations and the tendency of proteins to phase-separate into liquid condensates such as stress granules^17^. Interestingly, lipoic acid and LM have recently been identified as modulators of stress granule formation^11^. To investigate whether stress granule formation can be attenuated by LA or LM-mediated HDAC inhibition, A549 cells were treated with (*S*)-LA (no HDAC inhibition) or the HDAC inhibitors (*R*)-LA, (*R*/*S*)-LM and Vorinostat. After pre-incubation of cells with the compound, stress granule formation was induced using 30 min of arsenite treatment (1 mM). After treatment, cells were fixed for immunofluorescence detection of stress granules via the common stress granule marker protein G3BP1^21^. Importantly, only the HDAC inhibitors (*R*)-LA, (*R*/*S*)-LM, and Vorinostat, but not the HDAC-inactive (*S*)-LA led to a dose-dependent reduction of stress granule numbers per cell (Fig. 4a, b).

To understand, whether the purportedly antioxidant effects of lipoic acid play a role in the observed phenotypes, we performed assays to read out ROS levels in A549 cells exposed to stressors after drug pre-treatments. Levels of ROS induced by 2 h treatment of 200 µM tert-butyl hydroperoxide (tBuOOH) were significantly reduced to 60–70% by both (*S*)-LA and (*R*)-LA or the racemic form at concentrations of 100 µM (Fig. 4c, Supplementary Fig. 4). Another thiol-containing molecule and antioxidant, N-acetyl-cysteine (NAC), reduced oxidative stress levels to a similar extent as lipoic acid. Thus, while having differential activity on HDAC inhibition, protein acetylation, and stress granule formation, (*S*)-LA and (*R*)-LA showed comparable ROS buffering effects (Supplementary Fig. 5). The reduction of oxidative stress might be explained by the molecules’ capability of chelating ferrous or cuprous ions to suppress the Fenton reaction^22,23^. Scavenging of ROS by the molecules’ thiol groups could in theory play a role but the published literature concludes that this is kinetically irrelevant compared to enzyme-catalyzed ROS turnover^22–24^. Of note, physiological intracellular hydrogen peroxide concentrations are estimated to be in the low nanomolar range^24,25^ and 200 µM tBuOOH treatment, therefore, constitutes an extreme and highly non-physiological peroxide stress. Interestingly, in contrast to tBuOOH, exposing A549 cells for 30 min to 1 mM arsenite did not significantly induce ROS according to the CellRox assay (Fig. 4d, Supplementary Fig. 4). Thus, in the absence of extreme oxidative stress conditions as induced by 200 µM tBuOOH, neither NAC nor (*S*)-LA and (*R*)-LA pre-treatment had a significant ROS reducing effect in A549 cells. This finding supports the conclusion that the distinct effects of (*S*)-LA and (*R*)-LA on arsenite-induced stress granule formation are independent of the molecules’ potential as antioxidants, i.e., as metal ion chelators or scavengers of ROS. Considering the well-established role of HDACis in preventing arsenite-induced stress granule formation and the evidence for in-cellulo HDAC inhibition by R-Lipoic acid, we propose that enantioselective HDAC inhibition is a major contributor to differential phenotypes observed between (*S*)-LA and (*R*)-LA treatments.

## Discussion

Many of the metalloenzyme inhibitors widely used today, such as the angiotensin-converting enzyme inhibitor Captopril, contain thiols as metal chelating warheads. One of the most potent HDAC inhibitors, Romidepsin, features an intramolecular disulfide bond, which is intracellularly reduced to expose a thiol group that binds to the Zn^2+^-ion in the active site of HDACs. Both lipoic acid and lipoamide also feature an intramolecular disulfide bridge that is known to be readily reduced in cells^3^. Therefore, we speculated that LA and LM might target metalloproteins by one or both of the thiols functioning as a metal-chelating warhead. In line with this idea, chemoproteomic affinity profiling using immobilized lipoic acid identified Zn^2+^-dependent HDACs as the only proteins bound specifically by LA and LM. This does not exclude the possibility that other targets may exist, as pulldown experiments were only performed in MV4-11, SW620, and A549 cells, which may not express all potential target proteins. Of note, the affinity matrix was created by immobilizing (*R*/*S*)-LA via an amidation of its carboxylic acid group. Any target protein that may rely on an interaction with the negatively charged carboxy-group, would not score in the assay. Importantly, the same argument clearly opposes the published hypothesis that lipoic acid might inhibit HDACs akin to other nutritional short-chain fatty acids by zinc chelation via its carboxy group^26,27^. We confirmed HDAC binding and inhibition by recombinant enzyme activity assays, demonstrated HDAC target engagement in cells by nano-BRET assays, and showed HDAC substrate hyperacetylation as the result of HDAC inhibition by lipoic acid. Cellular HDAC inhibition by lipoic acid occurred at 10 to 100-fold higher concentrations compared to the in vitro recombinant HDAC inhibition assay. This might be explained by incomplete intracellular reduction, lower intracellular compound concentration, or metabolic conversion of lipoic acid. However, the determined target affinities and inhibitory concentrations (low two-digit micromolar range) are still well below the dose range commonly applied in phenotypic studies of lipoic acid (1–5 mM). They are also below the peak plasma concentration of about 0.5 mM in humans^10^. This suggests that HDAC inhibition occurs in vivo at sites with high lipoic acid exposure (e.g., the blood or intestine epithelial cells) and that HDAC inhibition may at least in part explain several of the previously described phenotypes observed in response to lipoic acid.

The mode of action of lipoic acid has often been attributed to its metal ion chelation, antioxidant, and ROS scavenging properties, as well as to its potential impact on mitochondrial metabolism and biogenesis^28,29^. However, increased mitochondrial metabolism should not result from a presumed increased availability of lipoic acid as an enzyme cofactor, because cells are capable of producing lipoic acid as needed. Considering a conservatively estimated 50 mM concentration of thiols in cells (mostly provided by glutathione), even intracellular concentrations of 0.5 mM lipoic acid would increase the availability of ROS scavenging thiols by only 1–2%^3^. Additionally, small molecule scavengers of ROS are considered kinetically irrelevant compared to the ROS turnover catalyzed by enzymes^22–24^. Antioxidant effects of lipoic acid might therefore rather be linked to cuprous or ferrous ion chelation, which prevents the metal ion-catalyzed creation of highly reactive hydroxyl radicals from the poorly reactive hydrogen peroxide (Fenton reaction)^22–24^. In line with that, both (*S*)-LA and (*R*)-LA buffered oxidative stress levels to 60–70% under extreme peroxide exposure (200 µM). In contrast, under non-stressed or arsenite-stressed conditions, lipoic acid did not significantly affect the oxidative stress levels in A549 cells. While metal ion chelation or ROS scavenging are physicochemical properties independent of lipoic acid stereochemistry, the enantiomer selective prevention of stress granule formation by (*R*)-LA argues for a specific mode of action.

In light of the data presented above, pan-HDAC inhibition by LA provides an attractive alternative way to explain many of the described cellular phenotypes of LA. For instance, our results relate the inhibition of stress granule formation by lipoic acid or LM to HDAC inhibition and the consequential hyperacetylation of stress granule proteins. Indeed, it has been shown that posttranslational modifications can regulate the phase separation behavior of proteins^30,31^. This includes protein acetylation^17,32,33^, possibly by neutralizing positive charges in intrinsically disordered regions, which are important for protein-RNA or protein–protein interactions^34^. For instance, the stress granule protein DDX3X showed increased acetylation upon lipoic acid or LM treatment akin to HDAC6 inhibitors (Fig. 3a) and the particular acetylation site has been linked to the regulation of DDX3X phase separation and stress granule maturation^17^.

Aberrant phase separation and maturation of stress granules is a hallmark of several neurodegenerative diseases such as ALS^35,36^. Interestingly, a recent screen of 1600 compounds^11^ identified lipoic acid and lipoamide as the most promising hits for the disruption of ALS-associated stress granules. However, the data could not explain the underlying mode of action. In line with reports of HDAC6 inhibitors that modulate the formation of stress granules^17,37^ and that are discussed as promising drug candidates to ameliorate certain disease phenotypes of neurological diseases^38,39^, our findings suggest that HDAC inhibition is at least a contributing mode of action of LA and LM. Thus, the current study adds to the notion that HDAC inhibitors are general modulators of liquid condensates. Such modulators are now increasingly explored for their therapeutic potential in a broad range of diseases termed condensatopathies^40^. Intriguingly, there is some overlap between the use of lipoic acid in clinical trials and those disease areas, where HDAC6 inhibition is a potential therapeutic strategy. These diseases mostly comprise neurologic pathologies such as ALS^11,41^ or peripheral neuropathy and neuropathic pain^5,9,42,43^. Strikingly, Ricolinostat, an HDAC inhibitor with a target selectivity profile similar to that of lipoic acid and Vorinostat^12^ (Supplementary Fig. 2f), is in clinical phase II trials for neuropathic pain – a condition for which lipoic acid is an approved medicine^7^. As we propose that HDAC inhibition is an important underlying mode of action, the current work provides a rationale for testing more advanced HDAC inhibitors in clinical trials for diseases, where lipoic acid showed promising effects. Vice versa, lipoic acid may be an alternative to designated HDAC inhibitors in diseases where the toxicity of current HDAC drugs is a concern.

Interestingly, no clinical trial has been conducted with LM yet. LM shows a slightly different target profile than lipoic acid, including HDAC10 inhibition, and is a somewhat more potent pan-HDAC inhibitor. Its effects on intracellular acetylation and prevention of stress granule formation are comparable to lipoic acid. One may speculate that LM could have better bioavailability than lipoic acid owing to its lower polarity and a lower propensity for degradation via beta-oxidation, which is one of the major metabolic routes of lipoic acid^44^. Therefore, it may be an advantageous alternative to lipoic acid.

## Methods

### Preparation of iC^12^

4-Azidobutanamine (1 μmol) was reacted with dimethylsulfoxide (DMSO)-washed N-hydroxysuccinimide (NHS)-activated (~20 μmol per ml beads) sepharose beads (1 ml) and triethylamine (20 μl) in DMSO (2 ml) on an end-over-end shaker overnight at room temperature in the dark. Aminoethanol (50 μl) was then added to inactivate the remaining NHS-activated carboxylic acid groups. After 1 h, the beads were washed with 40 ml DMSO. Alkyne-NHOTHP (1 μmol) was then clicked to the azide-functionalized beads via incubation in 1:1:2 (v/v/v) DMSO:tBuOH:H_2_O (2 ml total volume including beads), 0.1 mM tris(benzyltriazolylmethyl)amine (TBTA), 2 mM CuSO_4_ and 2 mM sodium ascorbate for 16 h at room temperature in the dark on the end-over-end shaker. The beads were then washed with 20 ml of 1:1:2 (v/v/v) DMSO:tBuOH:H_2_O, 30 ml of 50 mM EDTA in water, and 30 ml ethanol, then reacted with 10 mM HCl in EtOH (10 ml) for 16 h at room temperature in the dark. Beads were washed with 50 ml ethanol to yield iC, and stored at 4 °C in EtOH.

### Preparation of iQ^12^

Quisinostat (1 μmol) was reacted with DMSO-washed NHS-activated (~20 μmol per ml beads) sepharose beads (1 ml) and triethylamine (20 μl) in DMSO (2 ml) on an end-over-end shaker overnight at room temperature in the dark. Aminoethanol (50 μl) was then added to inactivate the remaining NHS-activated carboxylic acid groups. After 16 h, the beads were washed with 10 ml DMSO and 30 ml EtOH to yield iQ, stored at 4 °C in EtOH.

### Preparation of iL

Ethylenediamine (1 µmol, in DMSO) was reacted with DMSO-washed NHS-activated (~20 µmol/mL beads) sepharose beads (1 mL) and triethylamine (15 µL) in DMSO (2 mL) on an end-over-end shaker for 16 h at RT in the dark (TLC with Kaiser test staining was used to monitor successful conversion). Aminoethanol (50 µL) was then added to inactivate the remaining NHS-activated carboxylic acid groups. After 2 h on an end-over-end shaker at RT, the beads were washed with DMSO (4 × 10 mL) and resuspended in anhydrous DMF (2 mL total volume). HATU (10 µmol, 100 μL of 100 mM stock in DMF), racemic lipoic acid (12 µmol, 120 μL of 100 mM stock in DMSO), Hünig’s base (20 µmol, 100 μL of 200 mM stock in DMF) and triethylamine (20 μL) were then added and the beads were incubated at RT for 16 h on an end-over-end shaker. Next, the beads were washed twice with 10 mL DMF and thrice with 10 mL ethanol. Beads were stored at 4 °C in EtOH.

### Preparation of cell lysates for chemoproteomic assays

Cell line MV4-11 (ATCC: CRL-9591) was grown in RPMI 1640 medium (PAN Biotech). SW620 (from NCI60 panel), HeLa S3 (ATCC: CCL-2.2), and A549 (ATCC: CCL-185) were grown in DMEM medium (PAN Biotech). All media were supplemented with 10% FBS (PAN Biotech) and cell lines were internally tested for Mycoplasma contamination. Cells were lysed in lysis buffer (0.8% Igepal, 50 mM Tris-HCl pH 7.5, 5% glycerol, 1.5 mM MgCl_2_, 150 mM NaCl, 1 mM Na_3_VO_4_, 25 mM NaF, 1 mM DTT, supplemented with protease inhibitors (SigmaFast, Sigma) and phosphatase inhibitors (prepared in-house according to Phosphatase inhibitor cocktail 1–3 from Sigma-Aldrich)). The protein amount of cell lysates was determined by Bradford assay and adjusted to an Igepal concentration of 0.4% and protein concentration of 5 mg/mL.

### Chemoproteomic competition assays^12^

The cell lysate was pre-incubated with different doses of the small molecule of interest and a DMSO vehicle control for 1 h at 30 °C in an end-over-end shaker, followed by incubation with 18 µL affinity matrix (iL, iQ, or iC) for 30 min at 30 °C in an end-over-end shaker. To assess the degree of protein depletion from lysates by the affinity matrix, a second pulldown (PDPD) with fresh beads was performed using the unbound protein fraction from the vehicle control flow through. The beads were washed (1 × 1 mL of lysis buffer without inhibitors and only 0.4% Igepal, 2 × 2 mL of lysis buffer without inhibitors and only 0.2% Igepal), and captured proteins were denatured with 8 M urea buffer, alkylated with 55 mM chloroacetamide and digested with Trypsin according to standard procedures. The resulting peptides were desalted on a C18 filter plate (Sep-Pak® tC18 µElution Plate, Waters), vacuum dried, and stored at −20 °C until LC–MSMS measurement.

### LC_-_MS/MS measurement of chemoproteomic assays

Peptides were analyzed via LC–MS/MS on a Dionex Ultimate3000 nano HPLC coupled to an Orbitrap HF (Thermo Fisher Scientific) or either one of two Orbitrap Fusion Lumos mass spectrometers, operated via the Thermo Scientific Xcalibur software. Peptides were loaded on a trap column (100 μm × 2 cm, packed in-house with Reprosil-Gold C18 ODS-3 5 μm resin, Dr. Maisch, Ammerbuch) and washed with 5 μL/min solvent A (0.1% formic acid in HPLC grade water) for 10 min. Peptides were then separated on an analytical column (75 μm × 40 cm, packed in-house with Reprosil-Gold C18 3 μm resin, Dr. Maisch, Ammerbuch) using a 50 min gradient ranging from 4 to 32% solvent B (0.1% formic acid, 5% DMSO in acetonitrile) in solvent A (0.1% formic acid, 5% DMSO in HPLC grade water) at a flow rate of 300 nL/min.

The mass spectrometers were operated in data-dependent mode, automatically switching between MS1 and MS2 spectra. MS1 spectra were acquired over a mass-to-charge (m/z) range of 360–1300 m/z at a resolution of 60,000 (at m/z 200) in the Orbitrap using a maximum injection time of 10 ms (HF) or 50 ms (Lumos) and an automatic gain control (AGC) target value of 3e6 (HF) or 4e5 (Lumos). Up to 15 (HF) or 12 (Lumos) peptide precursors were isolated (isolation width of 1.2 Th for HF and Lumos2 and 1.2 for Lumos1, maximum injection time of 75 ms, AGC value of 1e5 for HF and 2e5 for Lumos), fragmented by HCD using 25% (HF) or 30% (Lumos) normalized collision energy (NCE) and analyzed in the Orbitrap at a resolution of 15,000 (Lumos2) or 30,000 (HF and Lumos1). The dynamic exclusion duration of fragmented precursor ions was set to 20 s (Lumos1) or 30 s (HF, Lumos2).

### Protein identification and quantification

Protein identification and quantification were performed using MaxQuant^45^ (v 1.6.1.0) by searching the LC–MS/MS data against all canonical protein sequences as annotated in the Swissprot reference database (v03.12.15, 20193 entries, downloaded 22.03.2016) using the embedded search engine Andromeda. Carbamidomethylated cysteine was set as a fixed modification and oxidation of methionine and N-terminal protein acetylation as variable modifications. Trypsin/P was specified as the proteolytic enzyme and up to two missed cleavage sites were allowed. The precursor tolerance was set to 10 ppm and fragment ion tolerance to 20 ppm. The minimum length of amino acids was set to seven and all data were adjusted to 1% PSM and 1% protein FDR. Label-free quantification^45^ and match between runs were enabled.

### Chemoproteomic competition assay data analysis

For the competition assays, relative binding was calculated based on the protein intensity ratio to the DMSO control for every single inhibitor concentration. EC_50_ values were derived from a four-parameter log-logistic regression with variable slope (constrain: bottom > 0). The obtained EC_50_ values were multiplied with a protein-dependent correction factor (cf), resulting in the apparent *K*_d_ value (*K*_d_^app^). The correction factor is determined by calculating the ratio of the protein intensity of two consecutive pulldowns of the vehicle control sample^13^. Targets of the inhibitors were annotated manually according to published procedures^12,46,47^. In brief, a protein was considered a target or interactor of a target if the resulting binding curve showed a sigmoidal curve shape with a dose-dependent decrease of binding to the beads. Additionally, the number of unique peptides and MSMS counts per condition were taken into account. Positive target binding across several independent experiments performed with different cell lysates further substantiated our confidence for a true positive drug-target binding event.

### HDAC-Glo assay

The experiments were performed according to ref. ^48^. HDAC6 and class I HDAC inhibition was tested using the HDAC-Glo™ I/II Assay and Screening System (G6421, Promega) with recombinant human HDACs (BPS Bioscience; HDAC1 cat. #50051; HDAC2 cat. #50002; HDAC3/NcoR2 complex cat. #50003; HDAC6 cat. #50006; HDAC8 cat. #50008). The assay was carried out in a 384-well plate (Corning 4512) format according to the manufacturer’s description. Drug dosing was performed with a D300e Digital Dispenser (Tecan). HDACs (7 ng/mL for HDAC1, 25 ng/mL for HDAC2, 200 ng/mL for HDAC3/Ncor2 complex, 100 ng/mL for HDAC6, 200 ng/mL for HDAC8) and inhibitors were incubated together at room temperature (RT) for 30 min. After the addition of the HDAC-Glo™ I/II reagent, plates were shaken (800 rpm orbital shaker, 30 s), centrifuged (300 *g*, 1 min), and incubated at RT for 30 min. Luminescence was detected with a CLARIOstar (BMG Labtech) plate reader. The luminescence signal was normalized with 100 μM SAHA-treated inhibition controls and uninhibited positive controls. pIC50 values were calculated from log(inhibitor) vs. normalized luminescence by nonlinear regression in GraphPad Prism.

### HDAC10 TR-FRET assay^48^

TR-FRET assays were performed in white 384-well plates (Corning 4512) using 50 mM HEPES pH 8.0, 150 mM NaCl, 10 mM MgCl_2_, 1 mM EGTA and 0.01% Brij-35 as a buffer. The concentrations of reagent in 15 μL final assay volume were 5 nM TwinStrep-GST-HDAC10, 25 nM “Tubastatin-AF647-Tracer” and 0.1 nM DTBTA-Eu^3+^-labeled Streptactin. Inhibitors with a D300e Digital Dispenser (Tecan). After drug dosing to the premixed assay reagents in buffer, plates were shaken (800 rpm orbital shaker, 30 s), centrifuged (300*g*, 1 min), and incubated at RT in the dark for 90 min. TR-FRET was measured with a CLARIOstar (BMG Labtech) plate reader, equipped with TR-FRET filters. Sample wells were excited with 100 flashes and fluorescence emission detected at 665 nm and 620 nm. FRET ratios were calculated from a 665 nm/620 nm ratio and normalized for each plate using 50 μM SAHA-treated inhibition controls and uninhibited positive controls. pIC50 values were calculated as described for the HDAC-Glo assay.

### HDAC6 and HDAC10 BRET assay^48^

For the production of transfected HeLa mono-clones stably expressing HDAC-nanoBRET fusion proteins of HDAC10 and HDAC6-catalytic domain 2 (HDAC6CD2), plasmids expressing a fusion of HDAC10 with nanoluciferase were obtained from Promega (N2170). HeLa cells (0.75 × 10^6^) were seeded in a 6 cm dish and were transfected with a mix of 10 μg plasmid and 3 μL Fugene in 200 μL OptiMEM after 24 h. The intracellular target engagement assay on HDAC10 and HDAC6CD2 was performed using the NanoBRET™ Target Engagement Intracellular HDAC Assay (Promega N2081 and N2090) as described by the kit manufacturer in a 96-well plate (Corning 3600) format with 2 × 10^4^ cells per well and a tracer concentration of 0.3 μM. Inhibitors were dosed with a D300e Digital Dispenser (Tecan). DMSO concentrations were normalized to 0.5% for all wells. After dosing, assay plates were shaken at 800 rpm and incubated at 37 °C for 2 h followed by measurement of 450 nm and 650 nm luminescence (80 nm bandwidth) at room temperature with a CLARIOstar (BMG Labtech) plate reader 2 min after NanoLuc substrate addition. BRET ratios were calculated from 650 nm/450 nm luminescence and normalized for each plate using 50 μM SAHA-treated negative controls and uninhibited positive controls. pIC50 values were calculated as described in the HDAC-Glo assay.

### DDX3X and α-tubulin acetylation detection by western blot

0.2 × 10^6^ HEK293T (ATCC: CRL-3216) cells were transfected with pcDNA 3.1-HA CBP plasmids (1 μg/well for the 6-well plate) by FuGENE. After 2 days, cells were treated with ACY-738, lipoic acid, and lipoamide for 7 h, then were washed by ice-cold PBS and lysed in Triton lysis buffer (50 mM Tris, pH 8.0, 150 mM NaCl, 1 mM EDTA, 0.1% TritonX-100 and complete EDTA-free protease inhibitors (Roche)) for analysis. To detect protein acetylation, 0.2 μM Trichostatin A and 5 mM nicotinamide were added to PBS for washing, and 10 μM trichostatin A, 10 mM nicotinamide, and 50 mM sodium butyrate were added to Triton lysis buffer. Samples were boiled for 10 min in SDS–PAGE sample buffer, and separated with 4–12% NuPAGE gels (Invitrogen). Proteins were transferred onto PVDF membranes (Immobilon-P, Millipore), probed with the primary antibodies (Anti-HDAC6, rabbit mAb, CST#7558, diluted 1:1000; anti-HA-Tag (C29F4) Rabbit mAb CST#3724, diluted 1:1000; anti-DDX3X (Millipore#09-860), diluted 1:1000; anti-acetyl-DDX3X produced in house^17^, diluted 1:1000, Monoclonal anti-α-tubulin antibody produced in mouse, Sigma-Aldrich Cat#T9026, diluted 1:2000; anti-acetyl-α-tubulin (Lys40) Monoclonal Antibody (6-11B-1), Catalog # 32-2700, Invitrogen, diluted 1:1000) overnight and secondary antibody for 1 h under 5% non-fat dry milk in TBS or 5% BSA, 0.1% Tween20 in TBS blocking conditions. HRP-based chemiluminescence was detected with Amersham Imager 680 using ECL western blotting reagent (GE Healthcare).

### Histone H4 and α-tubulin AcK western blot in A549 and HEK293T

0.3 × 10^6^ A549 and HEK293T cells were seeded to each well of 6-well plates at Day 0. On Day 2, cells were treated with drugs for 6 h. Then cells were harvested by Cell Lifter (CORNING:3008) and lysed by RIPA buffer (supplied with cOmplete™ Protease Inhibitor Cocktail). Protein concentration was determined by BCA assay and the same protein input amount for each condition was loaded onto gels. Proteins were transferred onto PVDF membranes (Immobilon-P, Millipore), and probed with specific primary antibodies overnight (AcK-H4 antibody: Anti-acetyl-histone H4 antibody, catalog#06-866, Sigma, diluted 1:1000; Actin: Pan-actin antibody Sigma-Aldrich Cat#SAB4502632, diluted 1:2000; RRID: AB_10746710; α-tubulin: Monoclonal anti- α -tubulin antibody produced in mouse, Sigma-Aldrich Cat#T9026; RRID: AB_477593, diluted 1:2000; AcK-α-tubulin: Acetyl- α-tubulin (Lys40) Monoclonal Antibody (6-11B-1), Catalog # 32-2700, Invitrogen, diluted 1:1000). Then the secondary antibody was added for 1 h under 5% non-fat dry milk in TBS blocking conditions. HRP-based chemiluminescence was detected with Amersham Imager 680 using ECL western blotting reagent (GE Healthcare).

### Global acetyl-lysine detection by Western blot

HeLa S3 was grown in DMEM medium (PAN Biotech) supplemented with 10% FBS (PAN Biotech) and treated with drugs (final concentration of 0.1% DMSO) for indicated periods. Protein lysates were generated by harvesting cells in lysis buffer (0.8% NP40, 50 mM Tris-HCl pH 7.5, 5% glycerol, 1.5 mM MgCl_2_, 150 mM NaCl, 1 mM Na_3_VO_4_, 25 mM NaF, 1 mM DTT and supplemented with protease inhibitors (SigmaFast, Sigma) and phosphatase inhibitors (prepared in-house according to Phosphatase inhibitor cocktail 1–3 from Sigma-Aldrich)). The protein amount of cell lysates was determined by Bradford assay. Samples were boiled for 10 min in SDS–PAGE sample buffer, and separated with 4–12% NuPAGE gels (Invitrogen). Proteins were separated by SDS-PAGE and electro-transferred onto PVDF membranes. Blots were kept in Tris-buffered saline, supplemented with 0.05% Tween (TBS-T) and 4% BSA for 1 h at room temperature and then incubated with primary antibody diluted in 1× TBS, 0.05% Tween and 4% BSA overnight at 4 °C. Following antibodies were used: Acetylated-Lysine Antibody (Cell Signaling Technology, #9441 S, polyclonal rabbit IgG, diluted 1:1000), and beta-Actin Antibody (Santa Cruz Biotechnology, #sc-47778, 0.2 mg/mL, monoclonal mouse IgG, diluted 1:500). After antibody incubation, blots were washed in TBS-T and probed with the corresponding fluorophore-conjugated secondary antibody (ODYSSEY donkey-anti-rabbit (#926-68023), goat-anti-mouse (#926-32210)) for 30 min at room temperature. Acquisition and quantification of the band fluorescence intensities were carried out with the Odyssey (Licor) imaging system and corresponding software (v 3.0.29). Intensities of proteins were normalized to input beta-Actin and further normalized to the control treatments to calculate the relative acetylation change.

### Lipoic acid effect on stress granule formation

0.03 × 10^6^ A549 cells were seeded at 4-chamber slides (Thermo, Nunc) and cultured at 37 °C overnight. Cells were treated with HDAC inhibitor SAHA and Lipoic acid or LM for 6 h, followed by 1 mM Arsenite for 30 min. Cells were fixed by 4% PFA and permeabilized by 0.5% Triton X-100/PBS. 10% Goat serum in PBS was used for blocking. After manually selecting an area with cell confluency of 50–80%, 16 pictures were taken randomly around the central point (ZEISS software in-built function). Stress granule marker G3BP1 (Aviva Systems Biology, ARP37713_T100) was visualized and quantified by ImageJ. Statistical analysis was performed with GraphPad Prism.

### CellRox^TM^ deep red assay for tBuOOH-induced ROS

A549 cells were maintained in DMEM (Sigma) supplemented with 10% fetal bovine serum (Pan Biotech). Cells were seeded onto a 24-well plate with a flat and clear bottom (Ibidi) 24 h before imaging. The CellRox assay was performed according to the product guidelines (ThermoFisher). Briefly, cells were treated with lipoic acid, N-acetyl-cysteine (NAC), or vector control (DMSO) for 1 h, followed by a 2 h treatment with 200 µM tert-butyl hydroperoxide, and then stained with 5 µM CellRox Deep Red for 1 h. Cells were washed twice with FluoroBrite DMEM (ThermoFisher) supplemented with 10% FBS before imaging. CellRox Deep Red signal intensity was measured on a Leica DMI 6000 B epifluorescent microscope with a Cy5 filter set. Mean signal intensity per cell was determined from 60 to 180 (on average ~125) cells per replicate. Significance was calculated by ANOVA with a post hoc Dunnett’s multiple comparisons test against the DMSO control (GraphPad).

### CellRox^TM^ deep red assay for ROS quantification

A549 cells were maintained in DMEM (Sigma) supplemented with 10% fetal bovine serum (Pan Biotech). Cells were seeded onto a 24-well plate with a flat and clear bottom (Ibidi) 24 h before imaging. The CellRox assay was performed according to the product guidelines (ThermoFisher). Briefly, cells were treated with 300 µM lipoic acid, LM, N-acetyl-cysteine, 200 µM tert-butyl hydroperoxide, or vector control (DMSO) for 1 h, and then stained with 5 µM CellRox Deep Red for 1 h with additional 1 mM arsenite treatment 30 min into staining where indicated. Cells were washed twice with FluoroBrite DMEM (ThermoFisher) supplemented with 10% FBS and Glutamax (ThermoFisher) before imaging. CellRox Deep Red signal intensity was measured on a Leica DMI 6000B inverted microscope with a Cy5 filter set. Mean signal intensity per cell was determined from 60 to 180 (on average ~100) cells per replicate. Significance was calculated by ANOVA with a post hoc Dunnett’s multiple comparisons test against the DMSO control (GraphPad).

### Statistics and reproducibility

All information on statistical tests is provided within the figure legends. All experiments resulting in figures and data provided in this manuscript were performed once.

### Reporting summary

Further information on research design is available in the Nature Portfolio Reporting Summary linked to this article.

## Supplementary information

Supplementary Information

Peer Review File

Description of Additional Supplementary Files

Supplementary Data 1

Reporting Summary

## Data Availability

The mass spectrometry proteomics data, including the used Swiss-Prot reference database and.pdfs from initial data analysis, have been deposited in the MassIVE proteomics database with the dataset identifier MSV000091758 (https://massive.ucsd.edu/ProteoSAFe/static/massive.jsp). Source data are provided in this paper.

## Source data

Source Data
