# Supplementary material for: Chemoproteomic target deconvolution reveals Histone Deacetylases as targets of (R)-lipoic acid
Source: Nat Commun. 2023 Jun 15;14:3548. doi: 10.1038/s41467-023-39151-8 (PMC10272112; doi:10.1038/s41467-023-39151-8)
Supplement: Supplementary file 1 — Supplementary Information [file 41467_2023_39151_MOESM1_ESM.pdf]

Supplementary Information

Chemoproteomic target deconvolution reveals Histone Deacetylases as targets of (*R*)-lipoic acid

Severin Lechner[1], Raphael R. Steimbach[2,3], Longlong Wang[4,5], Marshall L. Deline[6], Yun-Chien Chang[1], Tobias Fromme[6,7], Martin Klingenspor[6,7,8], Patrick Matthias[4,5], Aubry K. Miller[2,9], Guillaume Médard[1], Bernhard Kuster[1,9,10]

[1]*Chair of Proteomics and Bioanalytics, TUM School of Life Sciences, Technical University of Munich, Freising, Germany.*
[2]*Cancer Drug Development, German Cancer Research Center (DKFZ), Heidelberg, Germany.*
[3]*Biosciences Faculty, Heidelberg University, Heidelberg, Germany.*
[4]*Friedrich Miescher Institute for Biomedical Research, 4058 Basel, Switzerland.*
[5]*Faculty of Sciences, University of Basel, 4031 Basel, Switzerland.*
[6]*Chair of Molecular Nutritional Medicine, TUM School of Life Sciences, Technical University of Munich, Freising, Germany.*
[7]*EKFZ - Else Kröner Fresenius Center for Nutritional Medicine, Technical University of Munich, Freising, Germany*
[8]*ZIEL Institute for Food & Health, Technical University of Munich, Freising, Germany.*
[9]*German Cancer Consortium (DKTK), Heidelberg, Germany.*
[10]*Bavarian Center for Biomolecular Mass Spectrometry (BayBioMS), Technical University of Munich, Freising, Germany.*

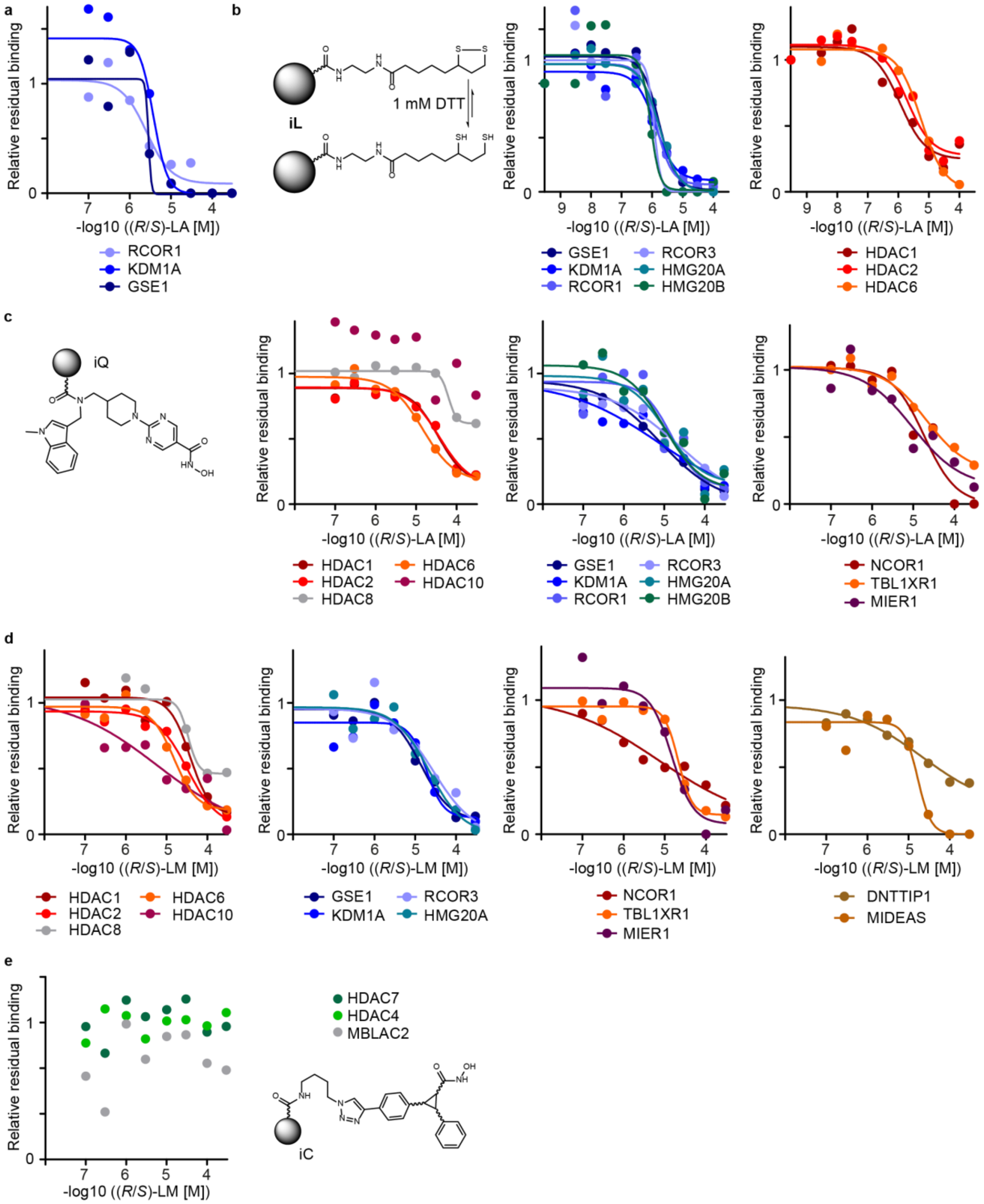

Supplementary Fig. 1 | Chemoproteomics identifies HDACs as targets of lipoic acid and lipoamide. a, Dose-response curves of HDAC CoREST complex partners from the LA-iL competition experiment in SW620 cell lysate. **b**, Dose-response curves of HDACs and CoREST complex partners from the LA-iL competition experiment in A549 cell lysate. **c**, Dose-response curves from the LA-iQ competition assay in SW620 cell lysate. HDAC interactor MIER1 and complex partners of the HDAC3 NCoR complex (NCoR1, TBL1XR1) as well as the HDAC1/2 CoREST complex (RCOR1/3, KDM1A, HMG20A/B, GSE1) show a dose-dependent reduction in affinity matrix binding. **d**, same as (c) but for lipoamide and including additional curves for MiDAC complex partners DNTTIP and MIDEAS. **e**, Competition assay for lipoic acid probing class IIa HDAC and MBLAC2 binding using established affinity matrix iC. Source data are provided as a Source Data file.

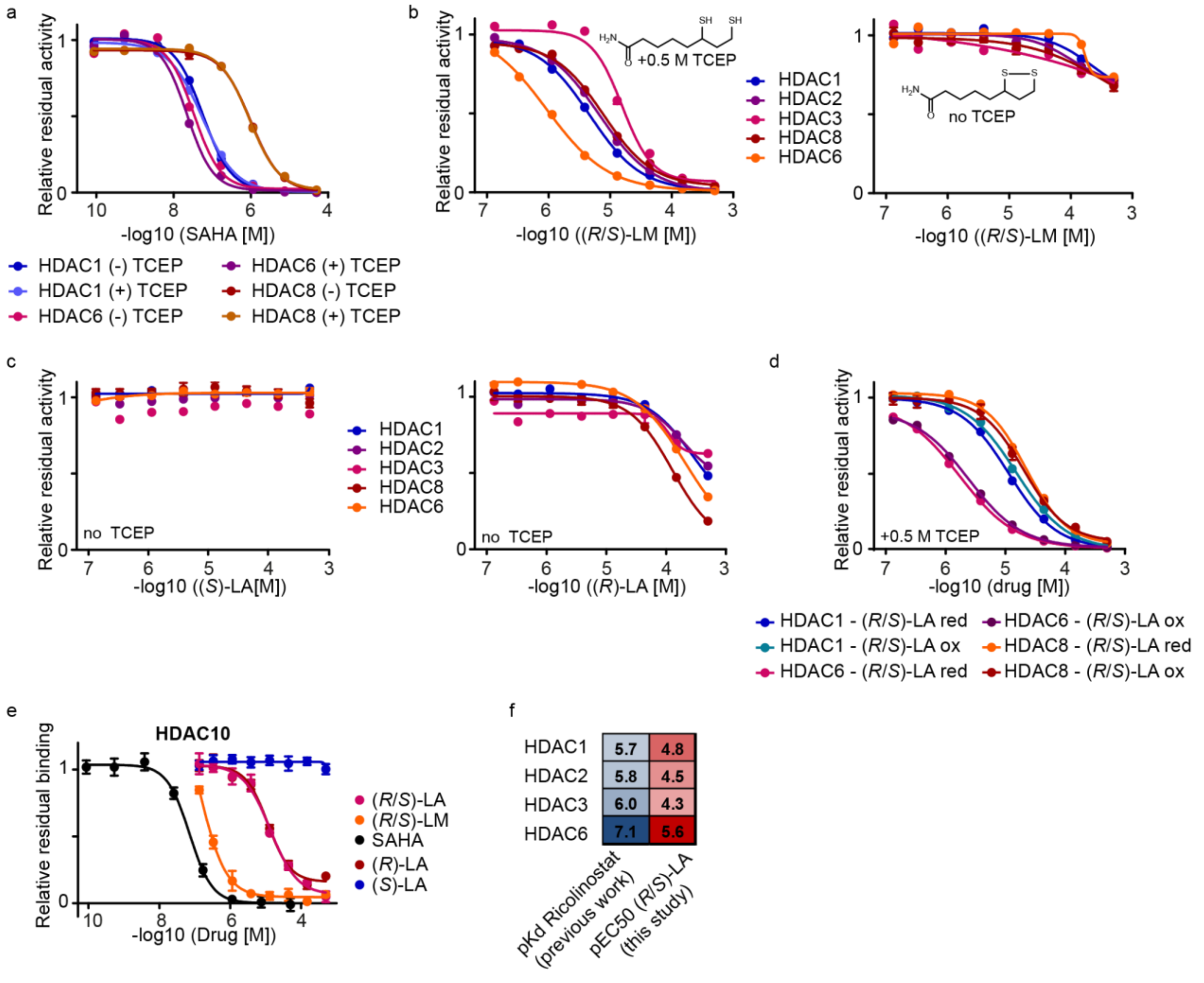

Supplementary Fig. 2|HDAC activity assays confirm the inhibitory effects of the reduced forms of (*R*)-lipoic acid, (*R*/*S*)-lipoic acid, and (*R*/*S*)-lipoamide. a, HDAC inhibition by SAHA (Vorinostat) in the Glo-assay setup is in the presence (+) or absence (-) of reducing agent TCEP (0.5 M) (n = 3 technical replicates for each drug dose, data are represented as mean value +/- SEM). **b,** influence of reducing agent TCEP (0.5 M) on Lipoamide mediated HDAC enzymatic inhibition via reduction and thiolane ring opening of the drugs (n = 3 technical replicates for each drug dose, data are represented as mean value +/- SEM). **c,** Dose-dependent effect of (*S*)- and (*R*)-lipoic acid on HDAC activity under non-reducing conditions (n = 3 technical replicates for each drug dose, data are represented as mean value +/- SEM). **d**, Comparison of effects of racemic reduced LA ((*R*/*S*)-LA red, i.e. dihydrolipoic acid) and oxidized (*R*/*S*)-LA on HDAC activity in the presence of reducing agent TCEP (0.5 M) (n = 3 technical replicates for each drug dose, data are represented as mean +/- SEM). **e**, FRET-based HDAC10 binding assay under reducing assay conditions (0.5 M TCEP) (n = 3 technical replicates for each drug dose, data are represented as mean value +/- SEM). **f**, lipoic acid pEC50 values from the Glo-HDAC activity assay are compared to the Ricolinostat pK_d^{app}s from ref.[1]. Source data are provided as a Source Data file.

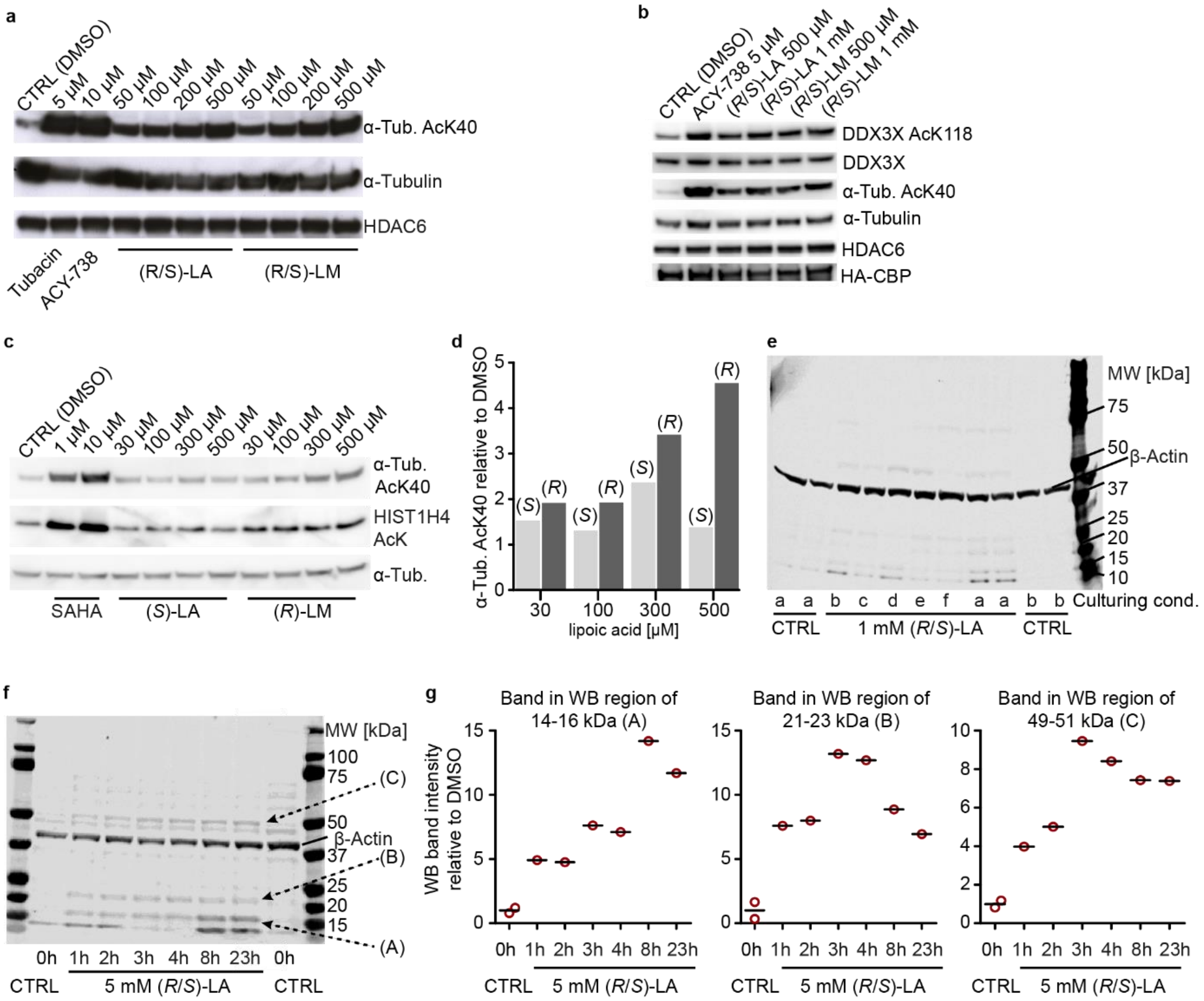

Supplementary Fig. 3|Lipoic acid and lipoamide treatment leads to hyperacetylation of HDAC substrate proteins. a, Western blot of CBP-HA (Acetyltransferase) transfected HEK293 cells that were incubated with HDAC6 inhibitor ACY-738, Tubacin, lipoic acid, or lipoamide for 7 h at indicated concentrations and probed for HDAC6 substrate protein acetylation site AcK40 of α-Tubulin as well as α-Tubulin and HDAC6 expression. **b**, Western blot of CBP-HA transfected HEK293 cells that were incubated with HDAC6 inhibitor ACY-738, lipoic acid, or lipoamide for 7 h at indicated concentrations and probed for HDAC6 substrate proteins and acetylation sites (Loading controls: HA-CBP and HDAC6). **c,** Western blot of HEK293T cells treated for 6 h with SAHA (Vorinostat), (*R*)-lipoic acid, or (*S*)-lipoic acid. **d,** comparison of dose-dependent effect of (*R*)-LA and (*S*)-LA treatment on α-tubulin AcK40 in HEK293 cells (quantification data from Supplementary Fig. 3c).**e**, Acetyl-lysine Western Blot of HeLa S3 cells cultured under various conditions and treated with 1 mM lipoic acid for 16 h. Culture conditions: a: DMEM (10% FBS), 95% confluency; b: DMEM (10% FBS), 70% confluency; c: IMDM (10% FBS), 70% confluency; d: supernatant of HeLa S3 cells after 3 d of culturing (old DMEM with 10% FBS), e: DMEM (10% FBS) plus 5 mM acetic acid, 70% confluency; f: DMEM (without FBS), 70% confluency. Biological replicates of condition 'a' treated with lipoic acid or vehicle control (DMSO) were used for quantification and plotting of Fig. 3c. **f** and **g**, Acetyl-lysine Western Blot of HeLa S3 cells treated with 5 mM (*R*/*S*)-lipoic acid for different periods (f) and intensity of bands (g) relative to control (DMSO, 0 h treatment) after normalization to β-actin loading control (n = 2 biologically independent samples for DMSO control CTRL, data of control is represented as mean value). Source data are provided as a Source Data file.

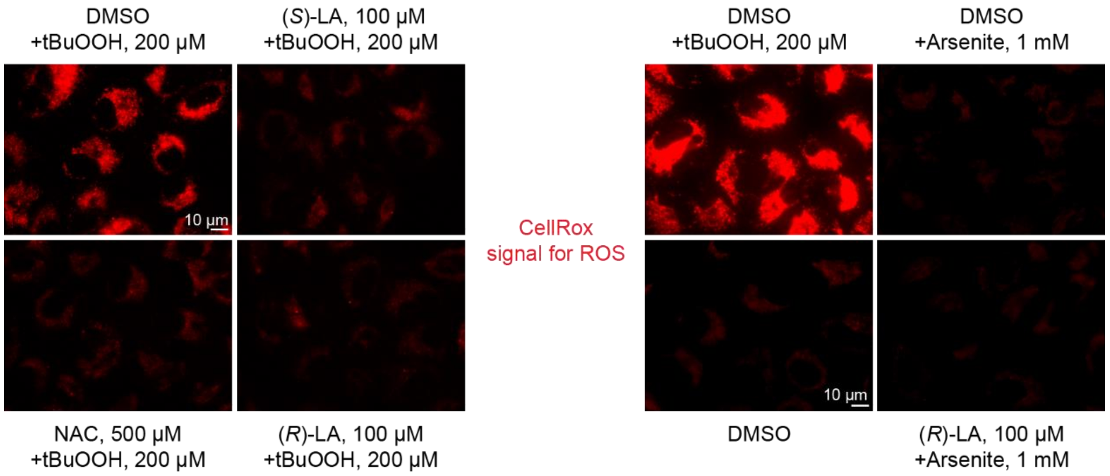

Supplementary Fig. 4|Small molecule ROS buffering effect under tertbutylhydroperoxide (tBuOOH) or arsenite stress. Cells were pretreated with drug molecules and then exposed to stress inducers tBuOOH or arsenite. ROS was quantified by microscopy via CellRox deep red signal intensity.

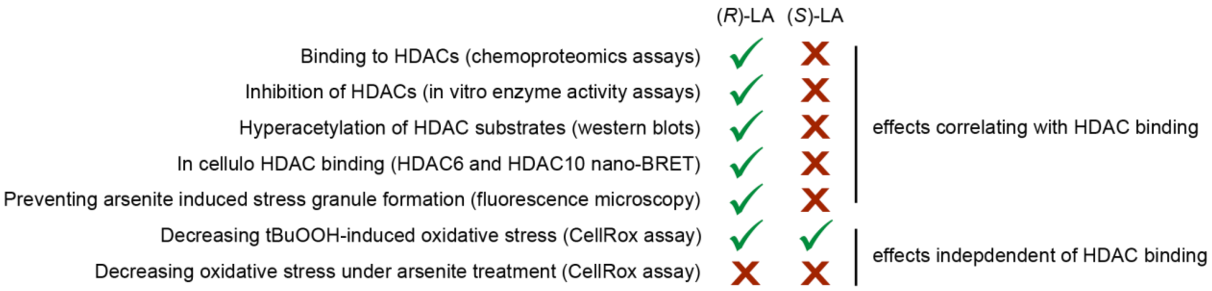

Supplementary Fig. 5|Overview of enantioselective effects of (*R*)-LA and of effects independent of the stereochemistry of LA. Assays used for the readout of the effects are provided in brackets.

Supplementary References

1. Lechner, S. *et al.* Target deconvolution of HDAC pharmacopoeia reveals MBLAC2 as common off-target. *Nat Chem Biol* **18**, 812-820 (2022).
