# Supplementary material for: Chemoproteomic target deconvolution reveals Histone Deacetylases as targets of (R)-lipoic acid
Source: Nat Commun. 2023 Jun 15;14:3548. doi: 10.1038/s41467-023-39151-8 (PMC10272112; doi:10.1038/s41467-023-39151-8)
Supplement: Supplementary file 3 — Description of Additional Supplementary Files [file 41467_2023_39151_MOESM3_ESM.docx]

File Name: Supplementary Data 1

Description: The table contains an overview of all pK_d_^app^ values derived from dose-response curves of chemoproteomic competition assays. Values are provided for direct targets (HDACs) and their interaction partners, which are assigned to their corresponding chromatin regulatory protein complex (CoREST, MiDAC, NCoR, or MIER). Further, information on the used affinity matrix and cell lysate is provided for each experiment.
