# Supplementary material for: Chemoproteomic target deconvolution reveals Histone Deacetylases as targets of (R)-lipoic acid
Source: Nat Commun. 2023 Jun 15;14:3548. doi: 10.1038/s41467-023-39151-8 (PMC10272112; doi:10.1038/s41467-023-39151-8)
Supplement: Supplementary file 5 — Reporting Summary [file 41467_2023_39151_MOESM5_ESM.pdf]

nature portfolio

Corresponding author(s): Bernhard Kuster

Last updated by author(s): May 23, 2023

Reporting Summary

Nature Portfolio wishes to improve the reproducibility of the work that we publish. This form provides structure for consistency and transparency in reporting. For further information on Nature Portfolio policies, see our Editorial Policies and the Editorial Policy Checklist.

Statistics

For all statistical analyses, confirm that the following items are present in the figure legend, table legend, main text, or Methods section.

n/a	Confirmed	
☐	☒	The exact sample size (n) for each experimental group/condition, given as a discrete number and unit of measurement
☒	☐	A statement on whether measurements were taken from distinct samples or whether the same sample was measured repeatedly
☐	☒	The statistical test(s) used AND whether they are one- or two-sided<br>*Only common tests should be described solely by name; describe more complex techniques in the Methods section.*
☒	☐	A description of all covariates tested
☐	☒	A description of any assumptions or corrections, such as tests of normality and adjustment for multiple comparisons
☐	☒	A full description of the statistical parameters including central tendency (e.g. means) or other basic estimates (e.g. regression coefficient) AND variation (e.g. standard deviation) or associated estimates of uncertainty (e.g. confidence intervals)
☐	☒	For null hypothesis testing, the test statistic (e.g. F, t, r) with confidence intervals, effect sizes, degrees of freedom and P value noted<br>*Give P values as exact values whenever suitable.*
☐	☒	For Bayesian analysis, information on the choice of priors and Markov chain Monte Carlo settings
☐	☒	For hierarchical and complex designs, identification of the appropriate level for tests and full reporting of outcomes
☒	☐	Estimates of effect sizes (e.g. Cohen's d, Pearson's r), indicating how they were calculated

Our web collection on statistics for biologists contains articles on many of the points above.

Software and code

Policy information about availability of computer code

Data collection	Thermo Scientific Xcalibur (v4.1.31.9) was used to collect mass spectrometry data. Odyssey Li-COR application software (v 3.0.29) was used for fluorescence based Western Blot scanning and quantification. Western blots read out by HRP-based chemiluminescence using the Amersham Imager 680. Fluorescence microscopy images determined for the stress granule experiment were acquired with a ZEISS-Wide Field Microscope. HDAC-Glo, HDAC10 TR-FRET, and BRET assay readout was performed with a CLARIOstar (BMG Labtech) plate reader.
Data analysis	MaxQuant (v 1.6.1.0) with embedded search engine Andromeda together with Swissprot reference database (v 03.12.15) were used to analyze the proteomics experiments. Internal R script based on the 'drc' package was used for initial data analysis and quality assessment of the chemoproteomics data. GraphPad PRISM (v5.01) was used to analyze and plot the data and to perform statistical tests on data. Quantification of HRP-based chemiluminescence was done with the help of Fiji-ImageJ (Version 1.8.0_112). For stress granule experiments, and ImageJ plugin “stress granule counter” (https://imagej.nih.gov/ij/plugins/stress_granule_counter/index.html) was used to quantify the stress granules. CellRox assay images were analyzed with FIJI (ImageJ version 1.53t).

For manuscripts utilizing custom algorithms or software that are central to the research but not yet described in published literature, software must be made available to editors and reviewers. We strongly encourage code deposition in a community repository (e.g. GitHub). See the Nature Portfolio guidelines for submitting code & software for further information.

Data

Policy information about availability of data

All manuscripts must include a data availability statement. This statement should provide the following information, where applicable:

- Accession codes, unique identifiers, or web links for publicly available datasets
- A description of any restrictions on data availability
- For clinical datasets or third party data, please ensure that the statement adheres to our policy

The mass spectrometry proteomics data, including the used Swiss-Prot reference database and .pdfs from initial data analysis, have been deposited to the MassIVE proteomics database with the dataset identifier MSV000091758. Source data are provide with this paper.

Research involving human participants, their data, or biological material

Policy information about studies with human participants or human data. See also policy information about sex, gender (identity/presentation), and sexual orientation and race, ethnicity and racism.

Reporting on sex and gender	No research involving human participants has been conducted
Reporting on race, ethnicity, or other socially relevant groupings	No research involving human participants has been conducted
Population characteristics	No research involving human participants has been conducted
Recruitment	No research involving human participants has been conducted
Ethics oversight	No research involving human participants has been conducted

Note that full information on the approval of the study protocol must also be provided in the manuscript.

Field-specific reporting

Please select the one below that is the best fit for your research. If you are not sure, read the appropriate sections before making your selection.

☒ Life sciences ☐ Behavioural & social sciences ☐ Ecological, evolutionary & environmental sciences

For a reference copy of the document with all sections, see nature.com/documents/nr-reporting-summary-flat.pdf

Life sciences study design

All studies must disclose on these points even when the disclosure is negative.

Sample size	No sample size or power calculation was performed. Regarding the CellRox assay, ten images per replicate was chosen arbitrarily to provide a sense of variation in the staining while preventing the timing of the imaging assay from exceeding two hours. Three replicates were chosen so that a statistics test could be performed.
Data exclusions	In Fig. 4c, one replicate of 100 uM (S)-LA was omitted due to obvious experimental errors
Replication	The number of replicates per condition is stated in the methods section and/or figure legends. Chemoproteomic dose dependent experiments were performed once per cell line.
Randomization	Images of stress granule were taken automatically by microscope.
Blinding	Blinding was not performed, since the type of experiments performed are not considered to be biased if not blinded.

Reporting for specific materials, systems and methods

We require information from authors about some types of materials, experimental systems and methods used in many studies. Here, indicate whether each material, system or method listed is relevant to your study. If you are not sure if a list item applies to your research, read the appropriate section before selecting a response.

Materials & experimental systems

n/a	Involved in the study
☐	☒ Antibodies
☐	☒ Eukaryotic cell lines
☒	☐ Palaeontology and archaeology
☒	☐ Animals and other organisms
☒	☐ Clinical data
☒	☐ Dual use research of concern
☒	☐ Plants

Methods

n/a	Involved in the study
☒	☐ ChIP-seq
☒	☐ Flow cytometry
☒	☐ MRI-based neuroimaging

Antibodies

Antibodies used

Anti Acetyl- α-tubulin (Lys40) Monoclonal Antibody ((6-11B-1), Catalog # 32-2700, Invitrogen). Anti-α-tubulin (Mouse monoclonal antibody, Sigma-Aldrich Cat#T9026; RRID: AB_477593). Anti-acetyl-histone H4 antibody (catalog#06-866, Sigma). Polyclonal rabbit Anti-actin-pan antibody (Sigma-Aldrich Cat#SAB4502632; RRID: AB_10746710). Acetylated-Lysine Antibody (Cell Signaling Technology, #9441S, polyclonal rabbit IgG). Beta-actin Antibody (Santa Cruz Biotechnology, #sc-47778, 0.2 mg/mL, monoclonal mouse IgG) . G3BP1 antibody (Aviva Systems Biology, ARP37713_T100). DDX3X antibody was established in the lab of one of the authors (10.1038/s41589-018-0180-7). 680LT Donkey anti-Rabbit IgG Secondary Antibody (IRDye®, #926-68023). 800CW Goat anti-Mouse IgG Secondary Antibody (IRDye®, #926-32210). Anti-HDAC6 (D2E5) Rabbit mAb CST#7558. Anti-HA-Tag (C29F4) Rabbit mAb CST#3724. Anti-DDX3X (Millipore, #09-860).

Validation

According to the manufacturers, antibodies are recommended for detection of analytes from human material per WB. Further, antibodies have been extensively used in the literature. DDX3X antibody was validated previously (10.1038/s41589-018-0180-7)

Eukaryotic cell lines

Policy information about cell lines and Sex and Gender in Research

Cell line source(s)

SW620 was provided by the NCI, as part of the NCI60 panel.
Following cell lines were acquired from ATCC: HEK293T (CRL-3216), A549 (CCL-185), HeLa S3 (CCL-2.2), and MV4-11 (CRL-9591).

Authentication

Multiplex human cell line authentication test was performed by multiplexion using SNP profiling for MV4-11. Other cell lines were not authenticated after being acquired.

Mycoplasma contamination

All cell lines were tested negative for mycoplasma contamination

Commonly misidentified lines
(See ICLAC register)

None.
