# Supplementary figures and images for: Chemoproteomic target deconvolution reveals Histone Deacetylases as targets of (R)-lipoic acid
Source: Nat Commun. 2023 Jun 15;14:3548. doi: 10.1038/s41467-023-39151-8 (PMC10272112; doi:10.1038/s41467-023-39151-8)

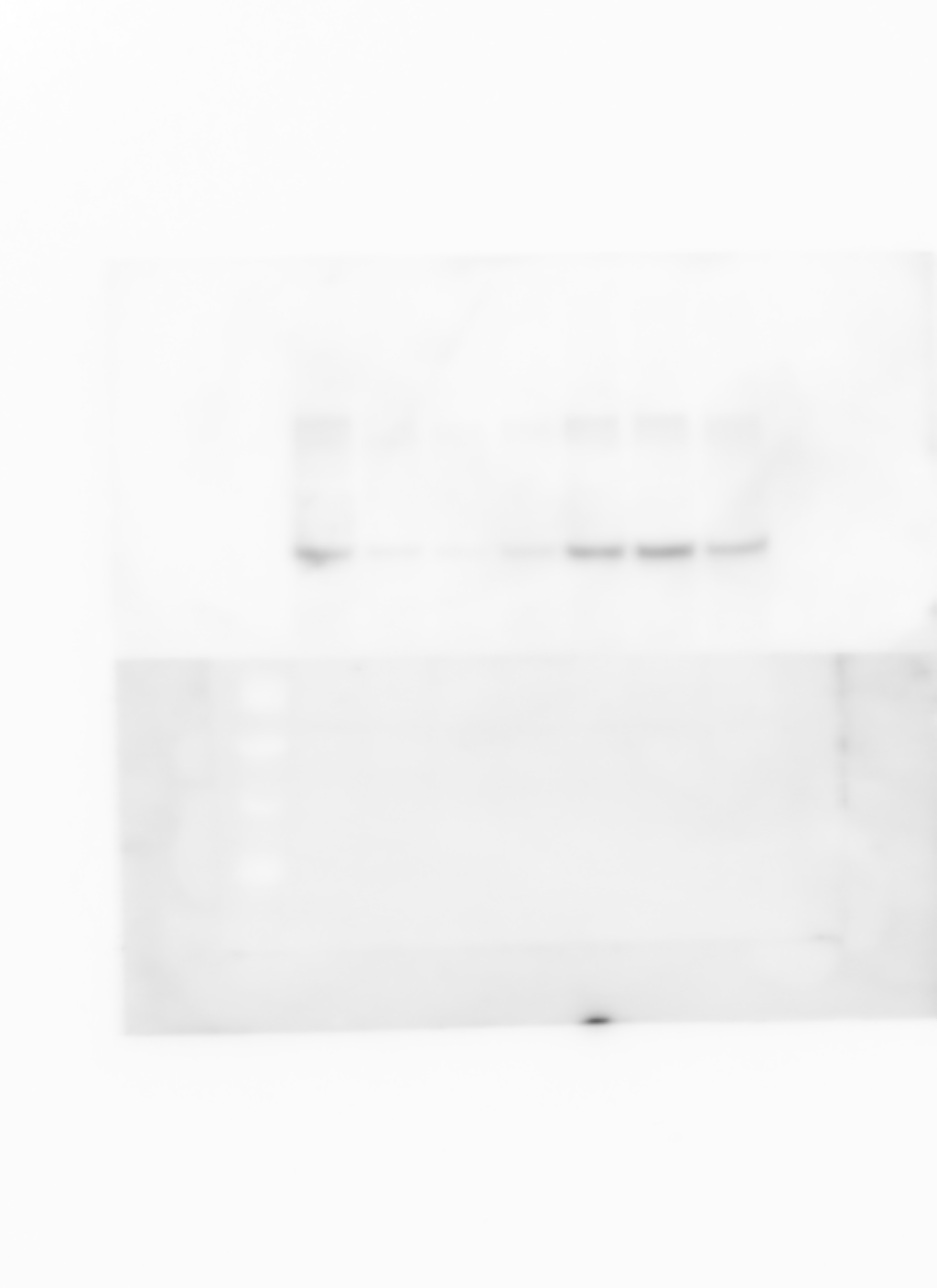

Supplement: Supplementary file 6 — Source Data [file 41467_2023_39151_MOESM6_ESM.zip › source data/Fig_3b_blots/Ac-a-tub uncropped.tif]

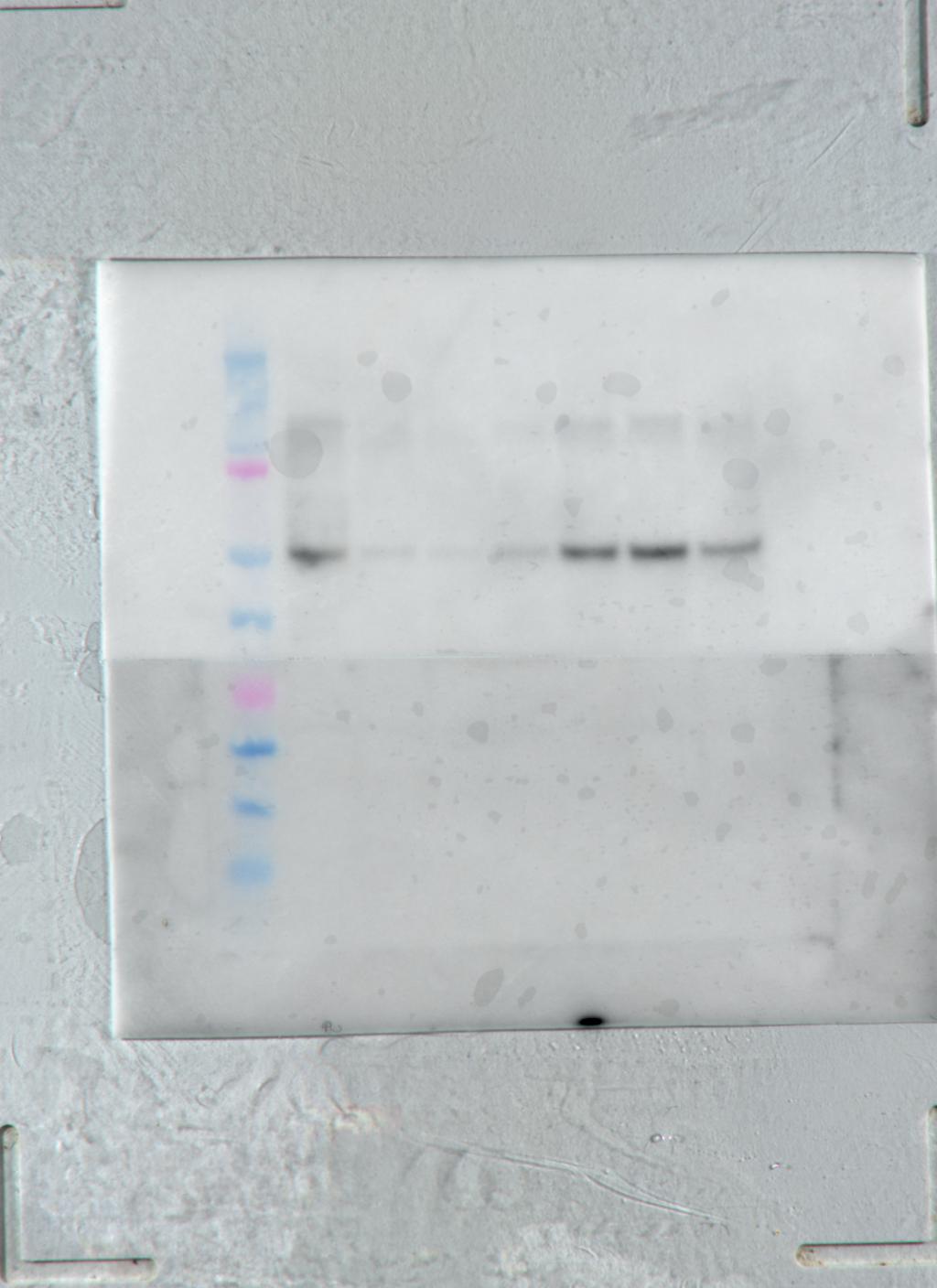

Supplement: Supplementary file 6 — Source Data [file 41467_2023_39151_MOESM6_ESM.zip › source data/Fig_3b_blots/Ac-a-tub uncropped_with marker.jpg]

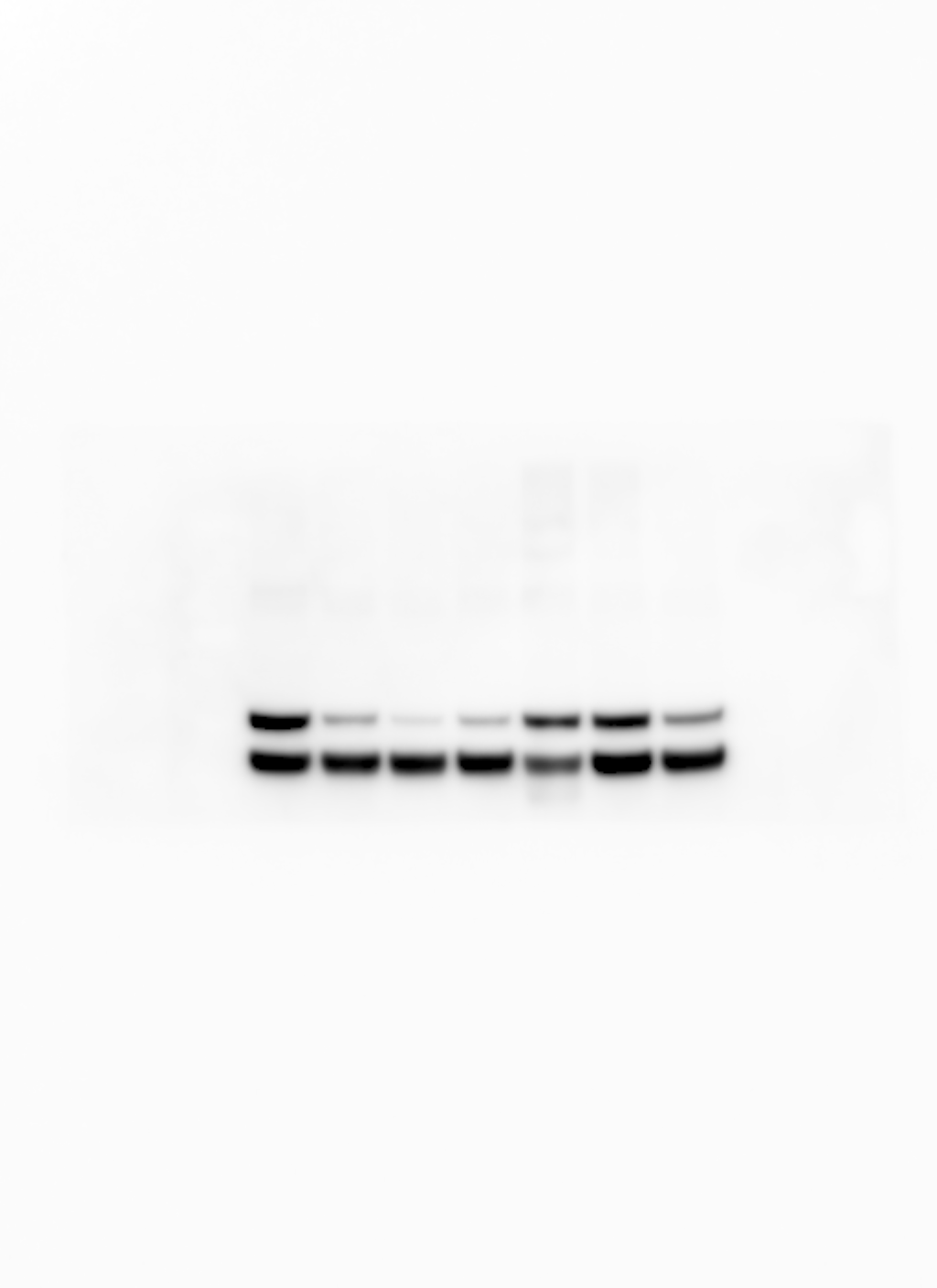

Supplement: Supplementary file 6 — Source Data [file 41467_2023_39151_MOESM6_ESM.zip › source data/Fig_3b_blots/actin uncropped.tif]

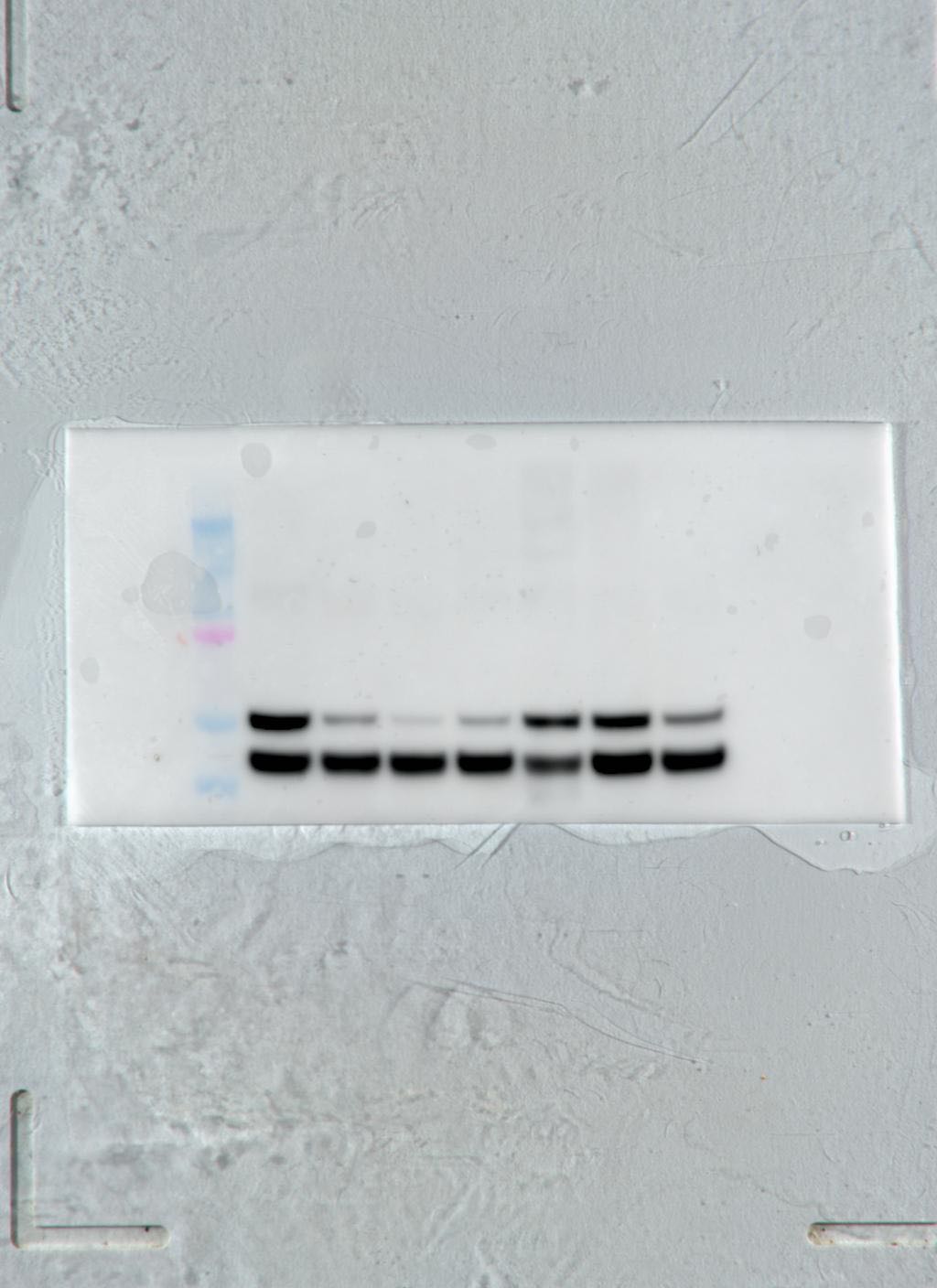

Supplement: Supplementary file 6 — Source Data [file 41467_2023_39151_MOESM6_ESM.zip › source data/Fig_3b_blots/actin uncropped_with marker.jpg]

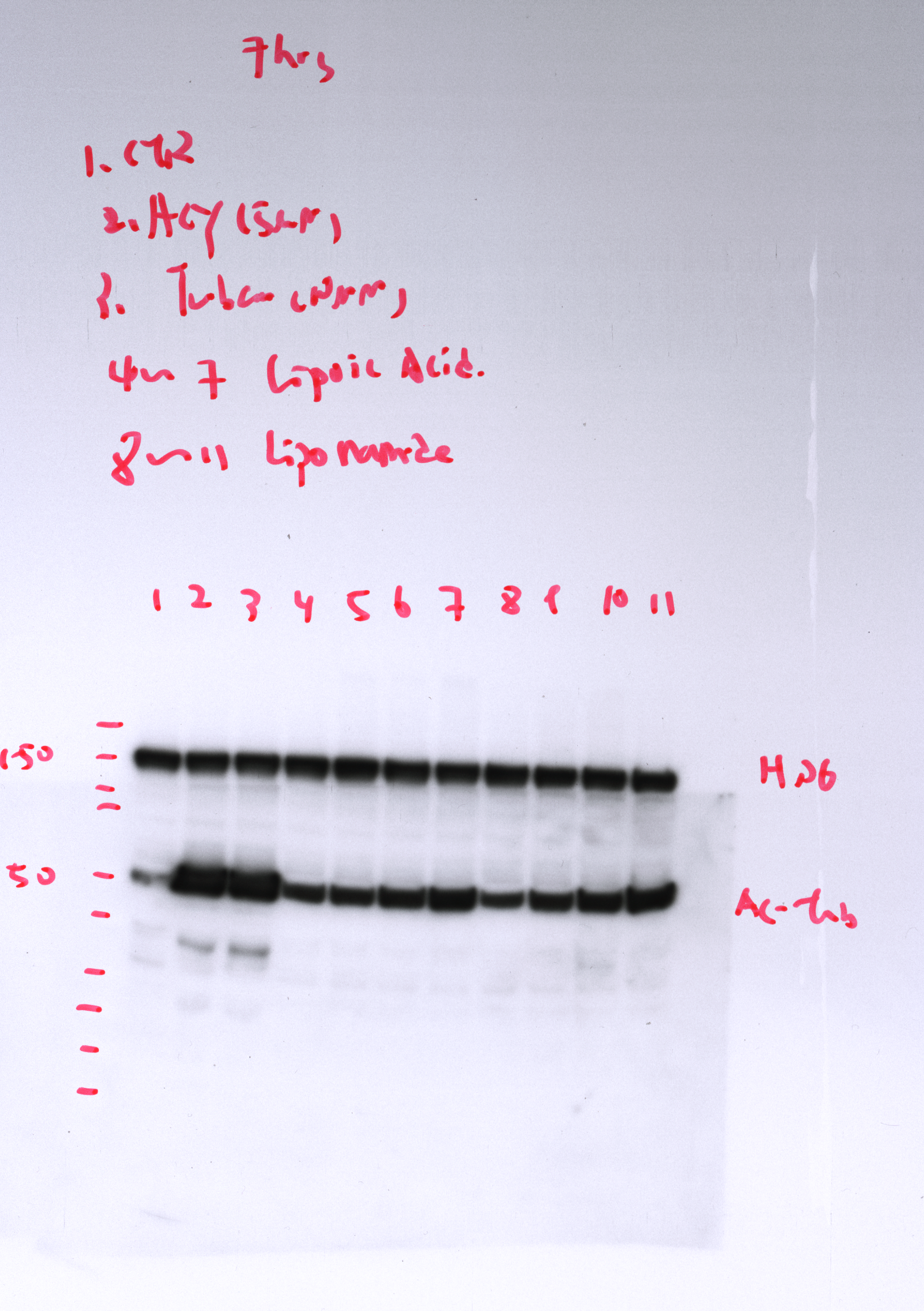

Supplement: Supplementary file 6 — Source Data [file 41467_2023_39151_MOESM6_ESM.zip › source data/Fig_S3a_blots/HDAC6 and Ac-aTub.tif]

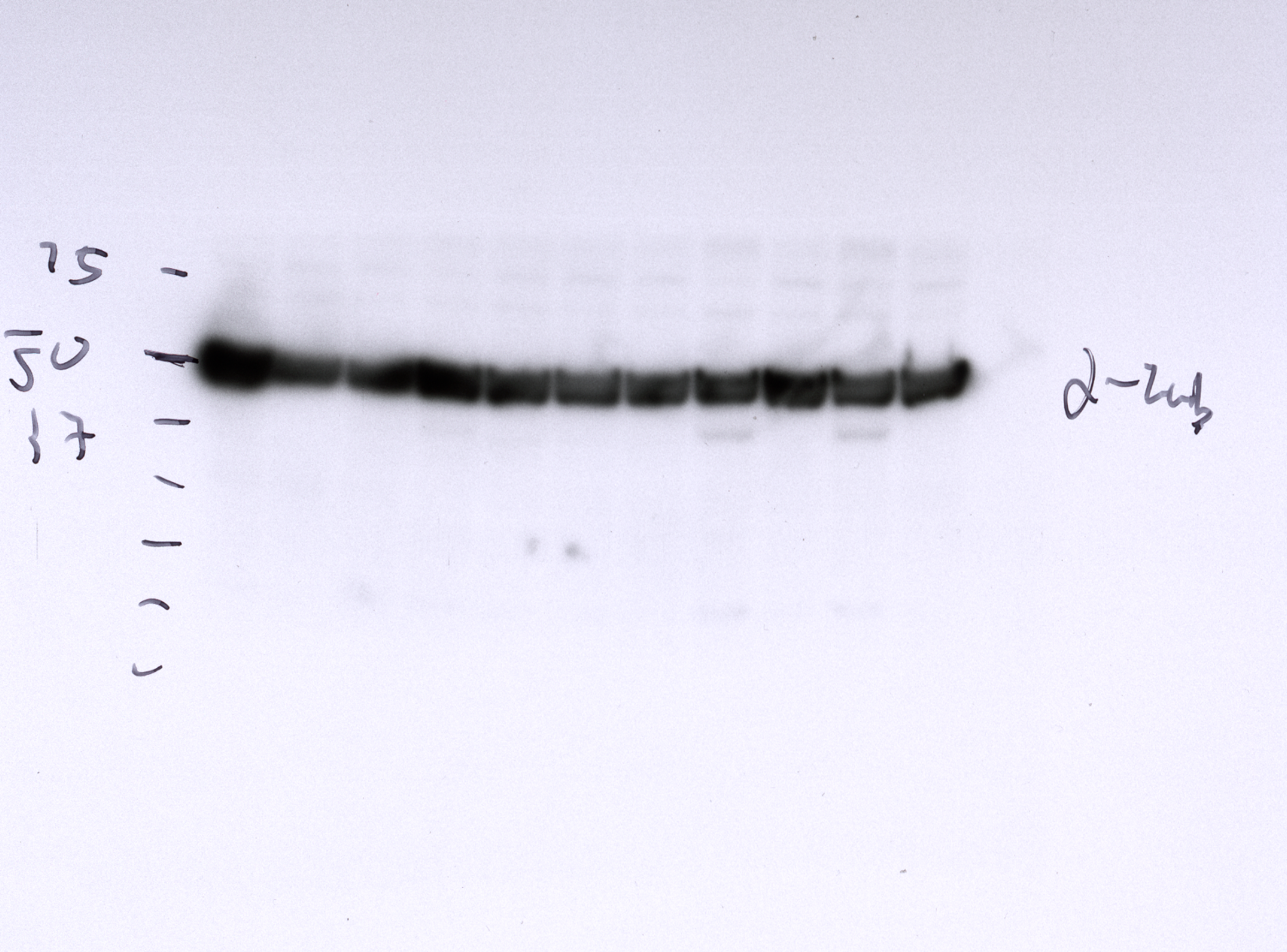

Supplement: Supplementary file 6 — Source Data [file 41467_2023_39151_MOESM6_ESM.zip › source data/Fig_S3a_blots/Tub.tif]

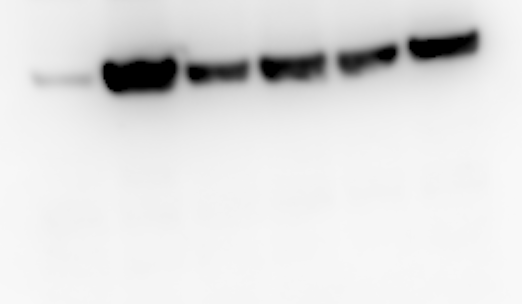

Supplement: Supplementary file 6 — Source Data [file 41467_2023_39151_MOESM6_ESM.zip › source data/Fig_S3b_blots/Ac-a-Tubulin.tif]

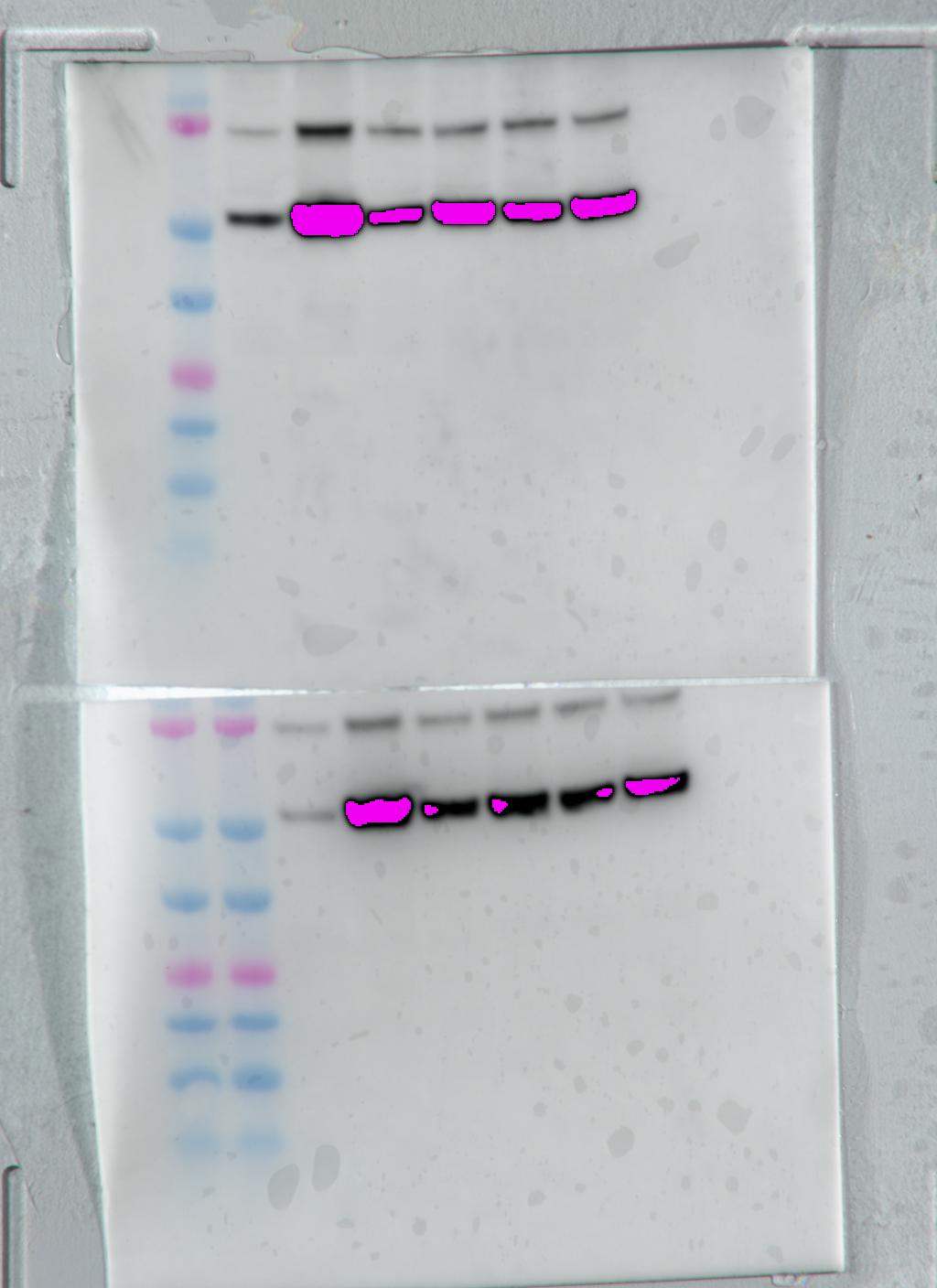

Supplement: Supplementary file 6 — Source Data [file 41467_2023_39151_MOESM6_ESM.zip › source data/Fig_S3b_blots/Ac-a-Tubulin_with marker.jpg]

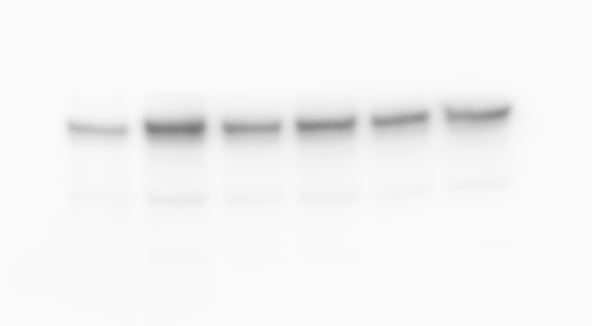

Supplement: Supplementary file 6 — Source Data [file 41467_2023_39151_MOESM6_ESM.zip › source data/Fig_S3b_blots/Ac-DDX3X.tif]

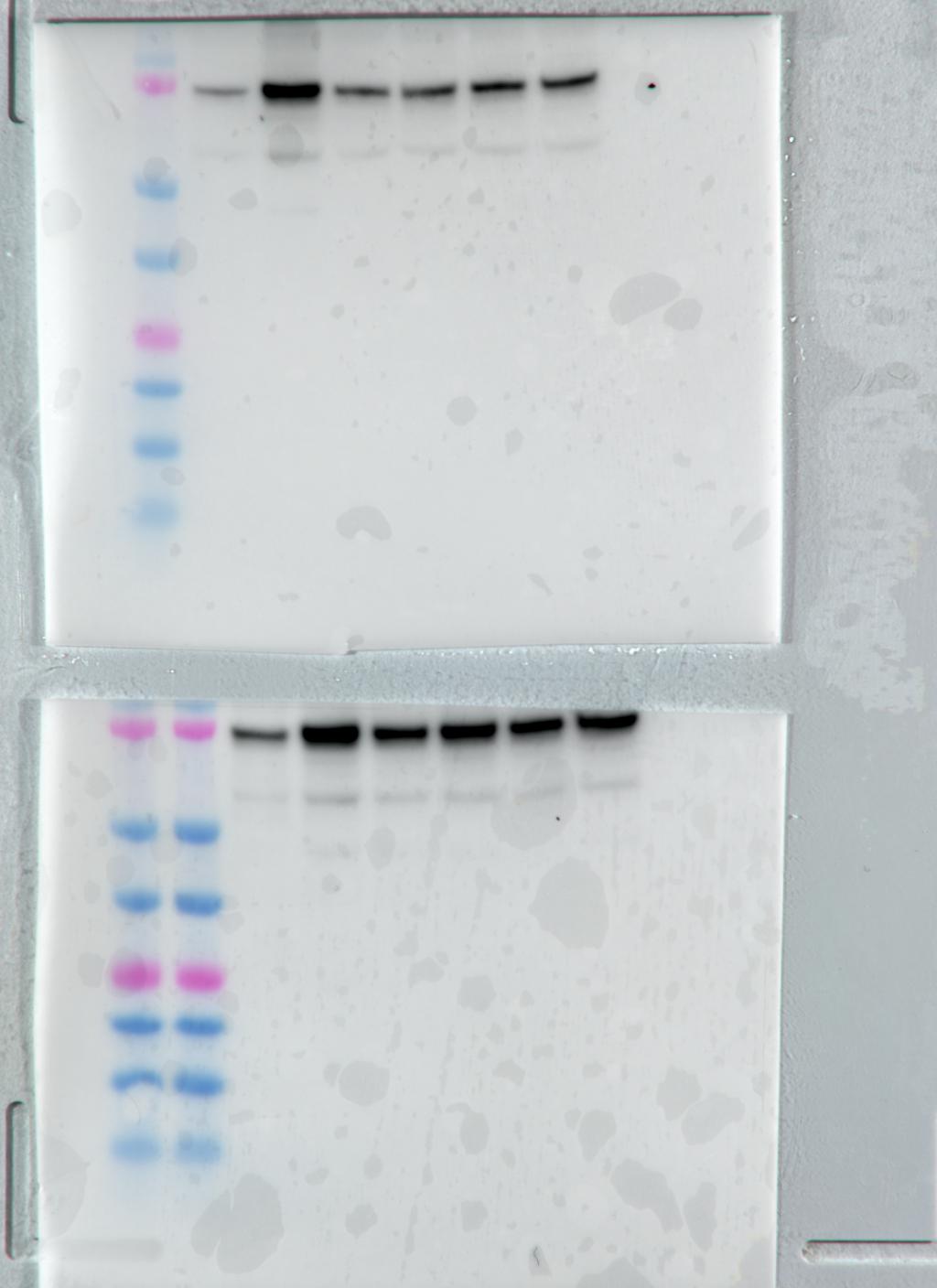

Supplement: Supplementary file 6 — Source Data [file 41467_2023_39151_MOESM6_ESM.zip › source data/Fig_S3b_blots/Ac-DDX3X_with marker.jpg]

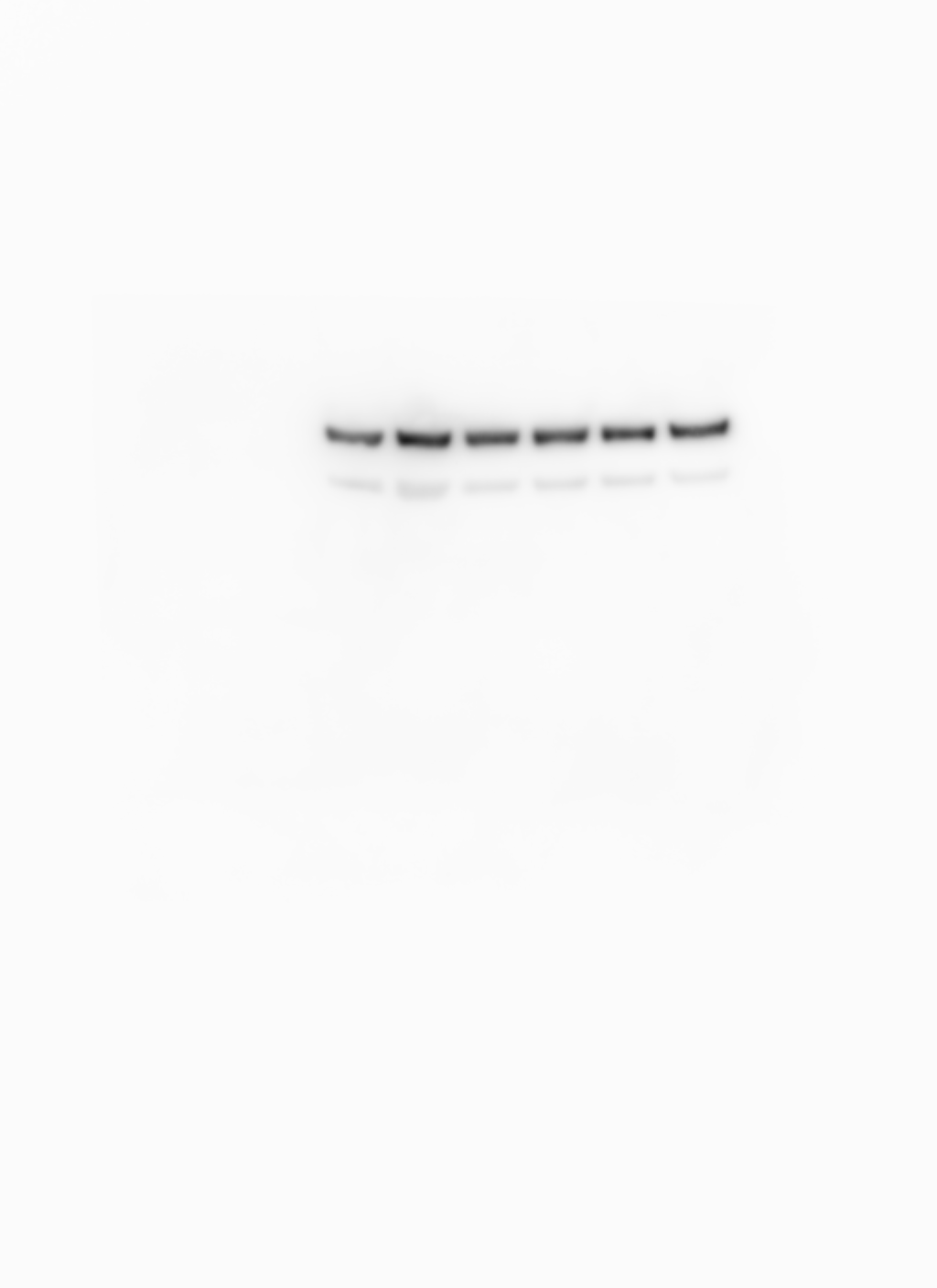

Supplement: Supplementary file 6 — Source Data [file 41467_2023_39151_MOESM6_ESM.zip › source data/Fig_S3b_blots/a-Tubulin.tif]

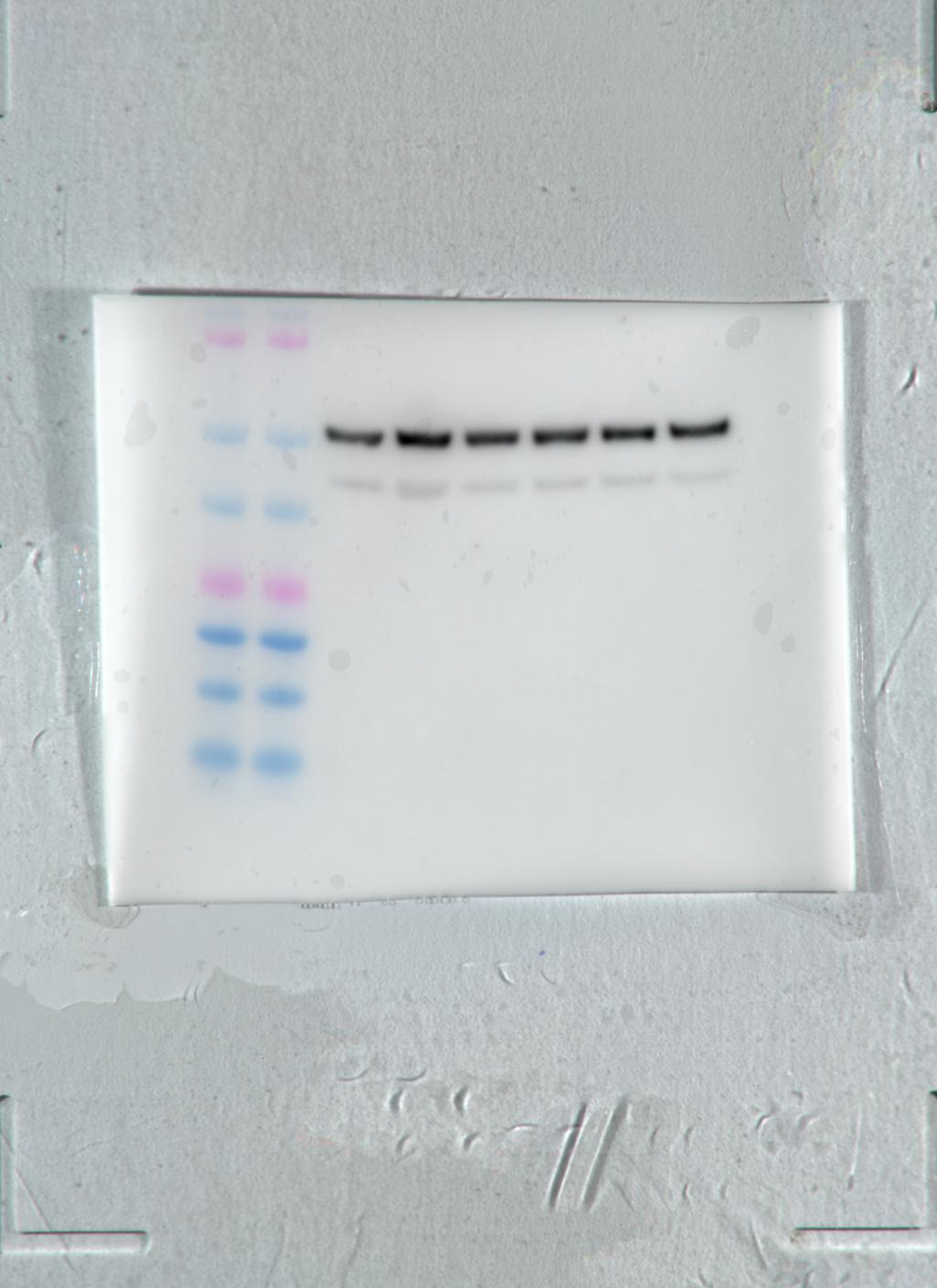

Supplement: Supplementary file 6 — Source Data [file 41467_2023_39151_MOESM6_ESM.zip › source data/Fig_S3b_blots/a-Tubulin_with marker.jpg]

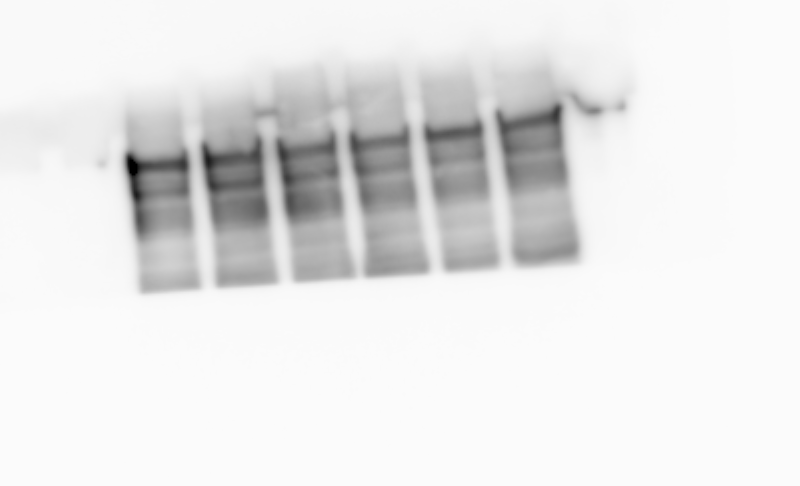

Supplement: Supplementary file 6 — Source Data [file 41467_2023_39151_MOESM6_ESM.zip › source data/Fig_S3b_blots/HA-CBP.tif]

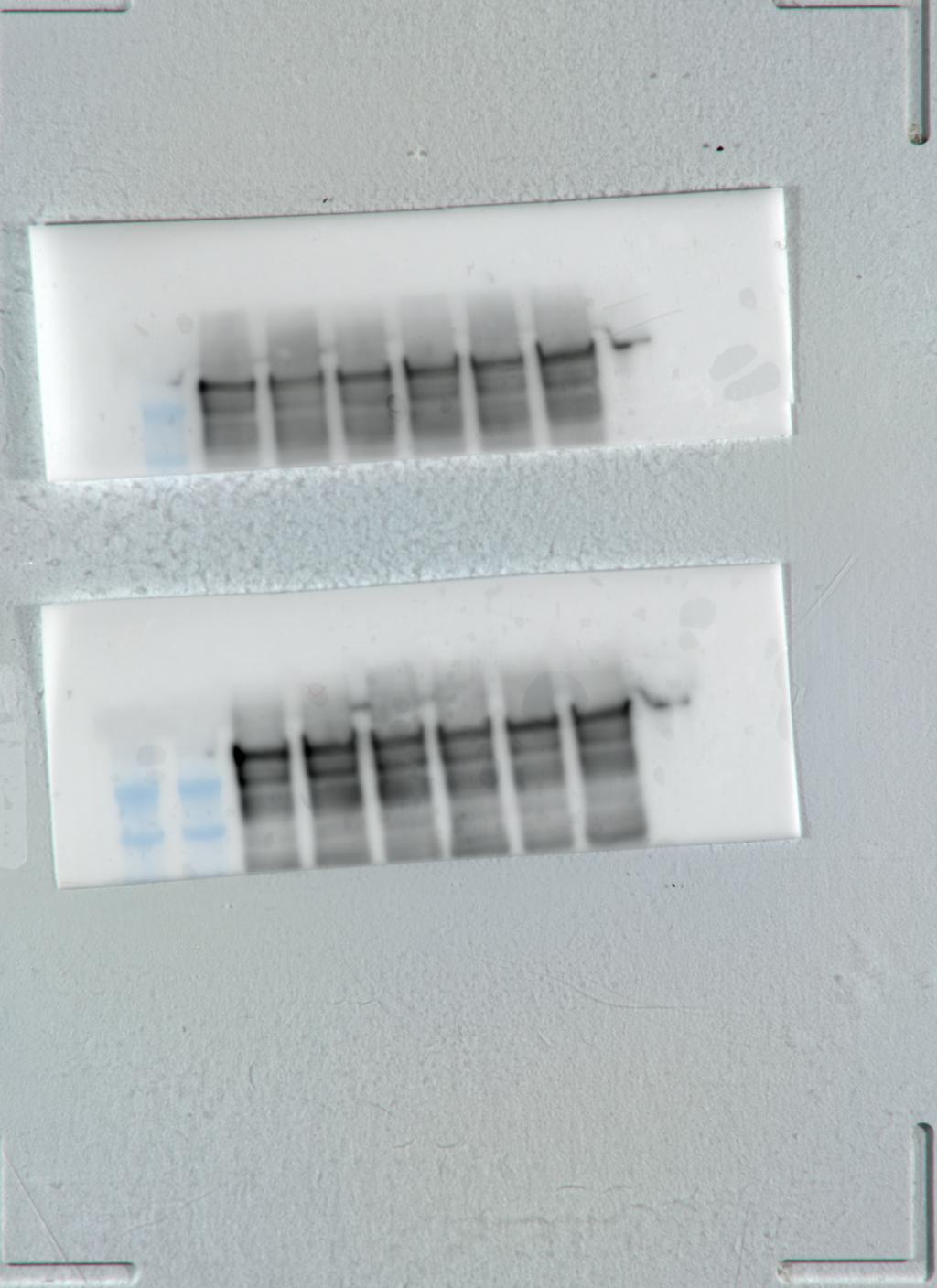

Supplement: Supplementary file 6 — Source Data [file 41467_2023_39151_MOESM6_ESM.zip › source data/Fig_S3b_blots/HA-CBP_with marker.jpg]

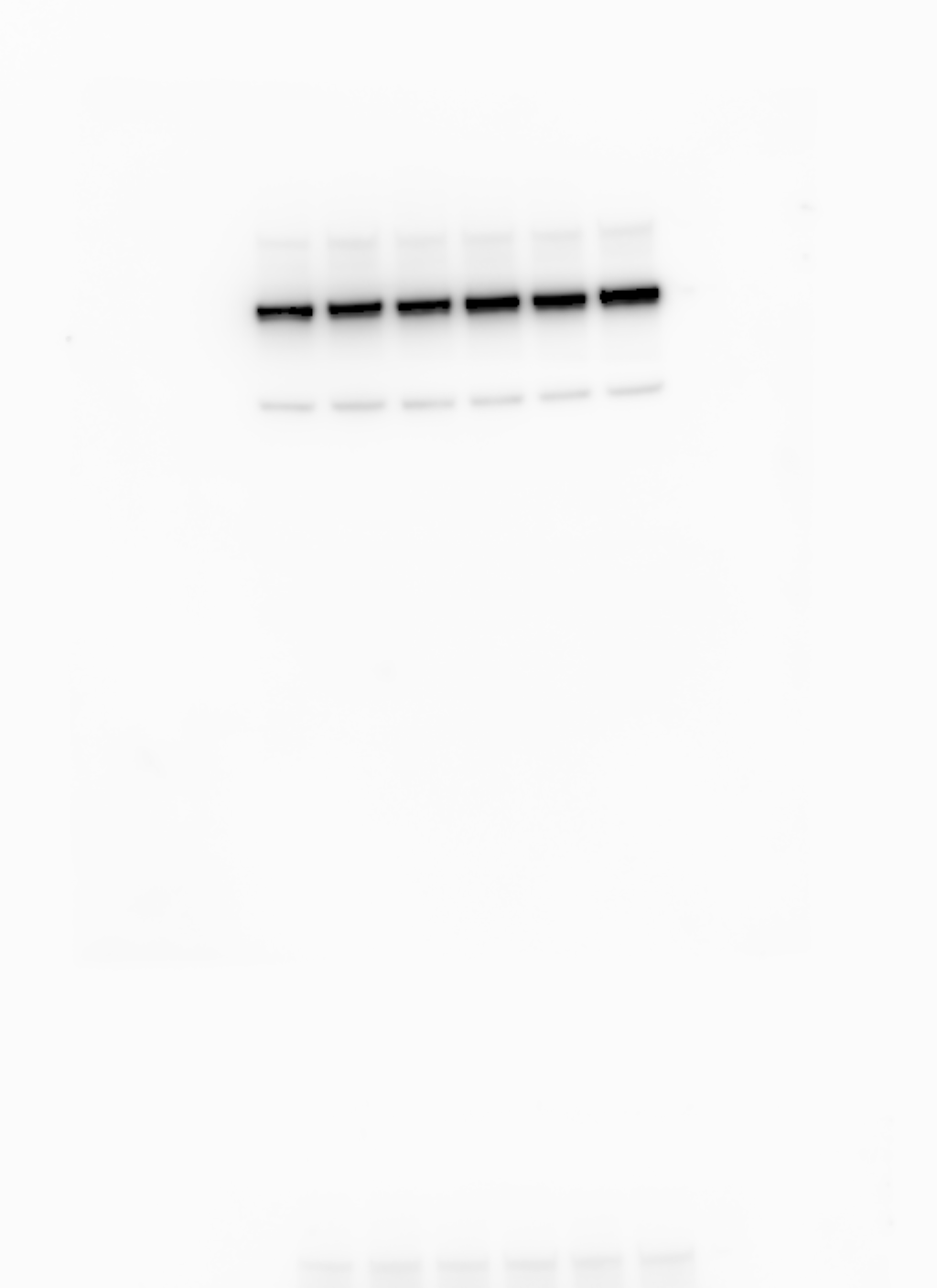

Supplement: Supplementary file 6 — Source Data [file 41467_2023_39151_MOESM6_ESM.zip › source data/Fig_S3b_blots/HDAC6_and_DDX3X.tif]

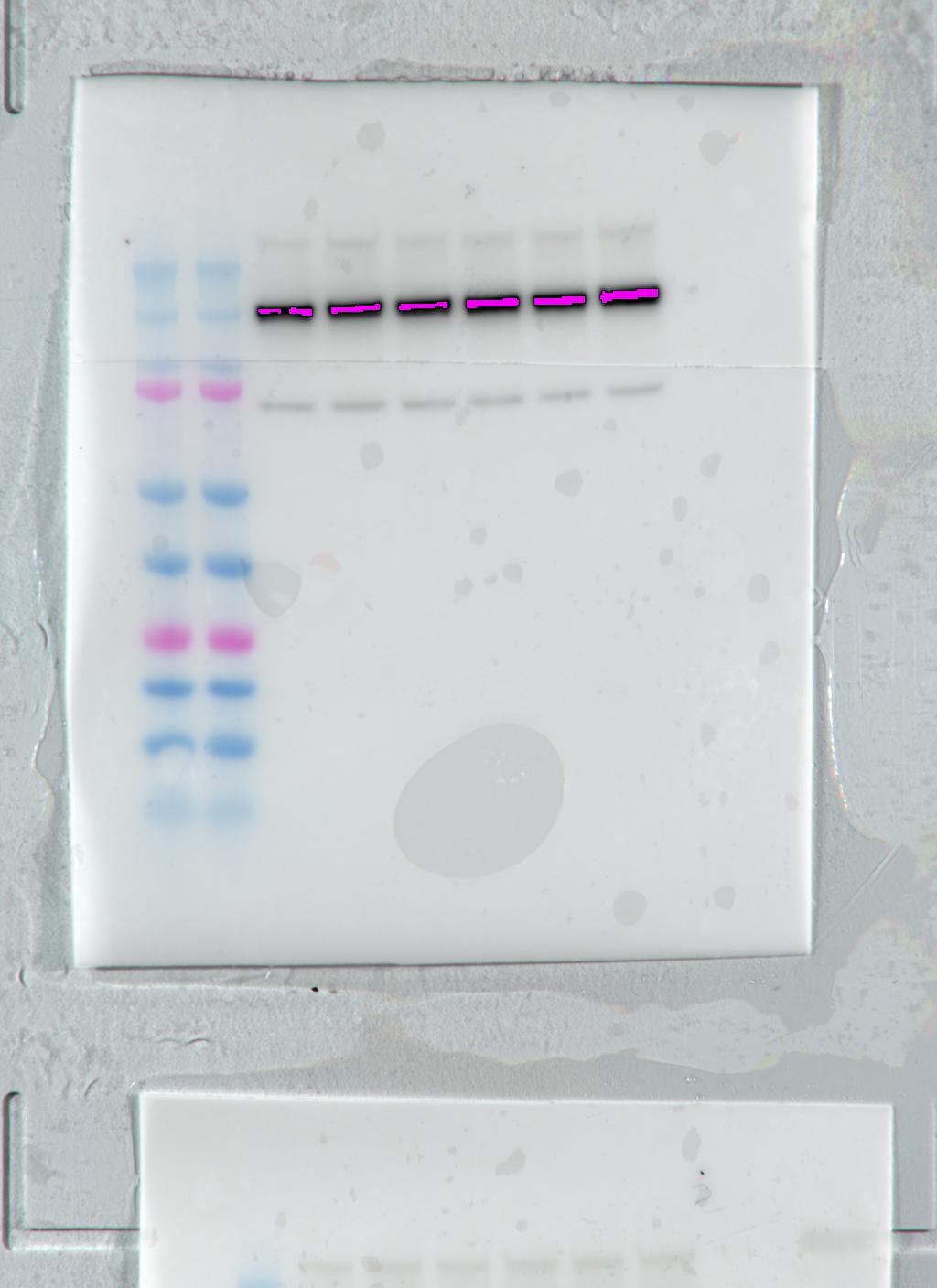

Supplement: Supplementary file 6 — Source Data [file 41467_2023_39151_MOESM6_ESM.zip › source data/Fig_S3b_blots/HDAC6_and_DDX3X_with marker.jpg]

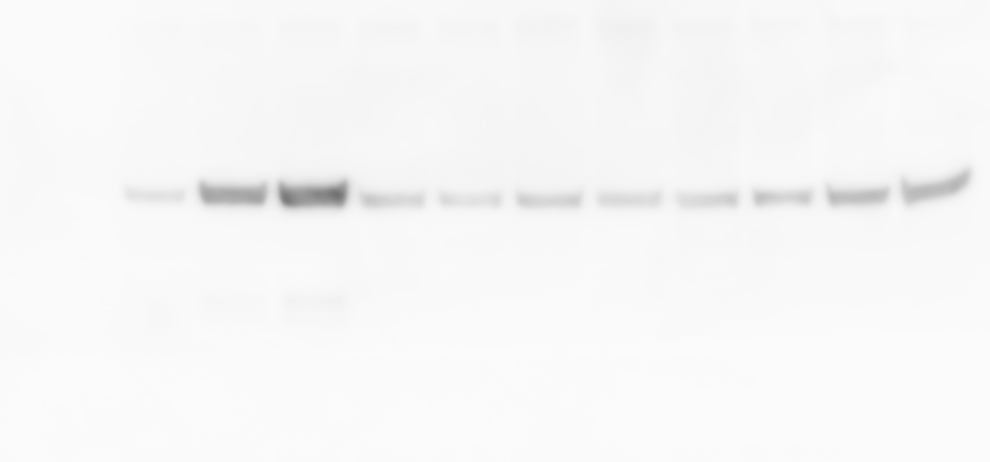

Supplement: Supplementary file 6 — Source Data [file 41467_2023_39151_MOESM6_ESM.zip › source data/Fig_S3c_blots/Ac-a-Tubulin.tif]

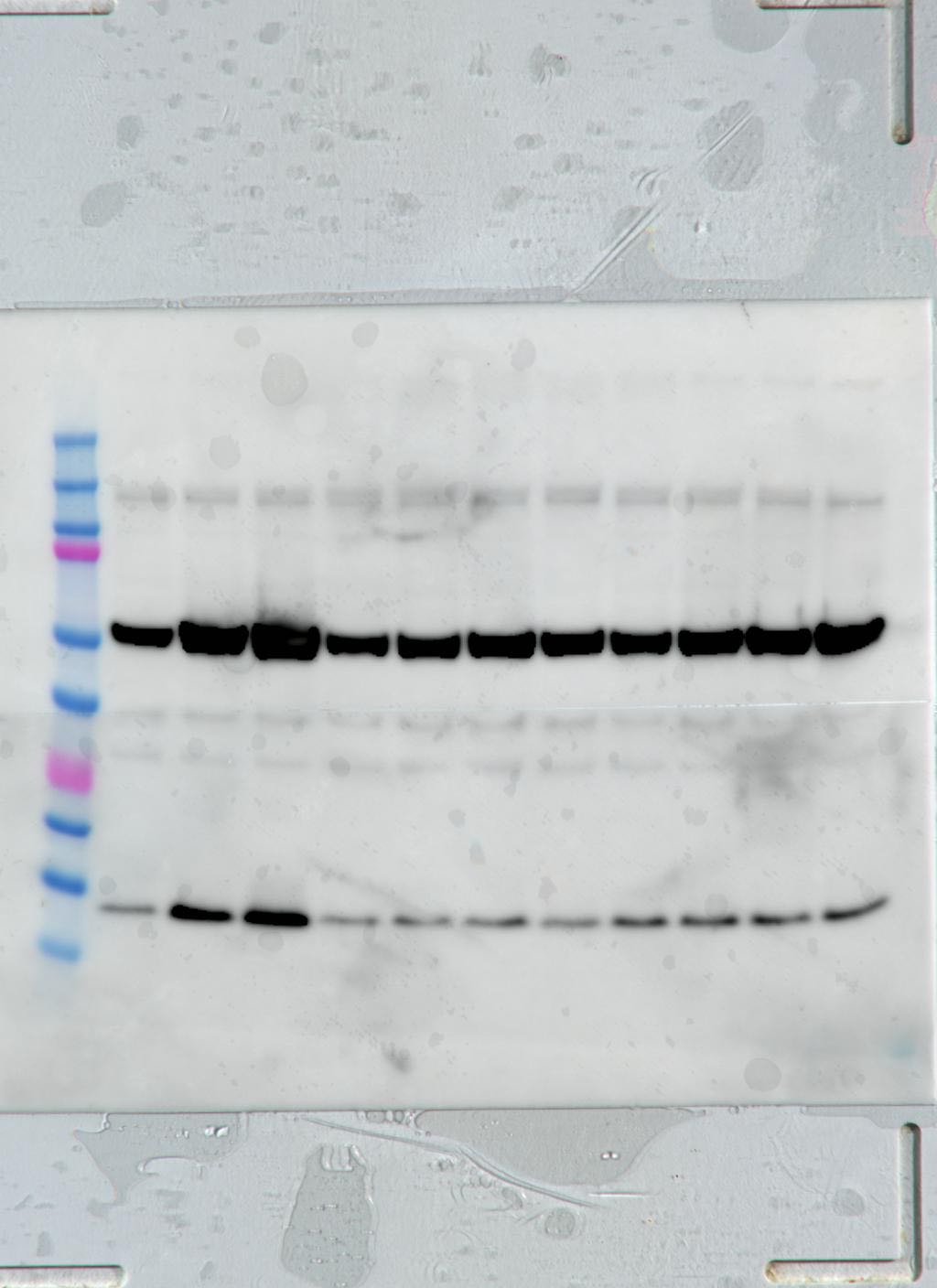

Supplement: Supplementary file 6 — Source Data [file 41467_2023_39151_MOESM6_ESM.zip › source data/Fig_S3c_blots/Ac-a-Tubulin_and_ AcH4_with marker.jpg]

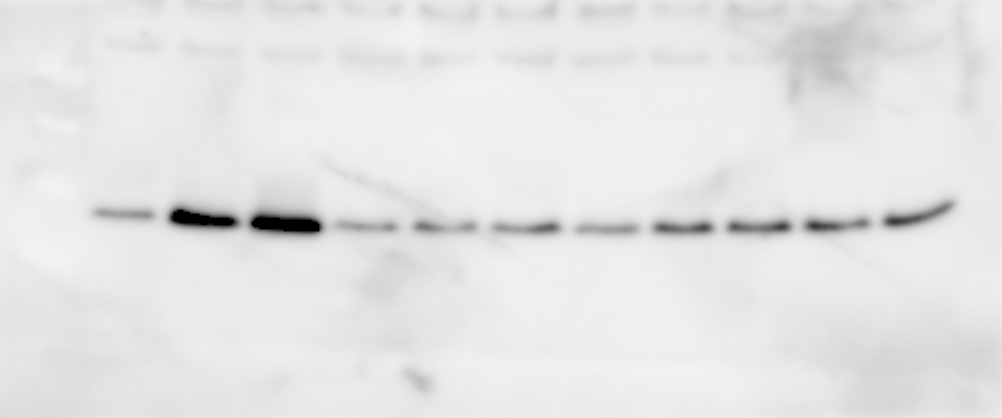

Supplement: Supplementary file 6 — Source Data [file 41467_2023_39151_MOESM6_ESM.zip › source data/Fig_S3c_blots/AcH4 all.tif]

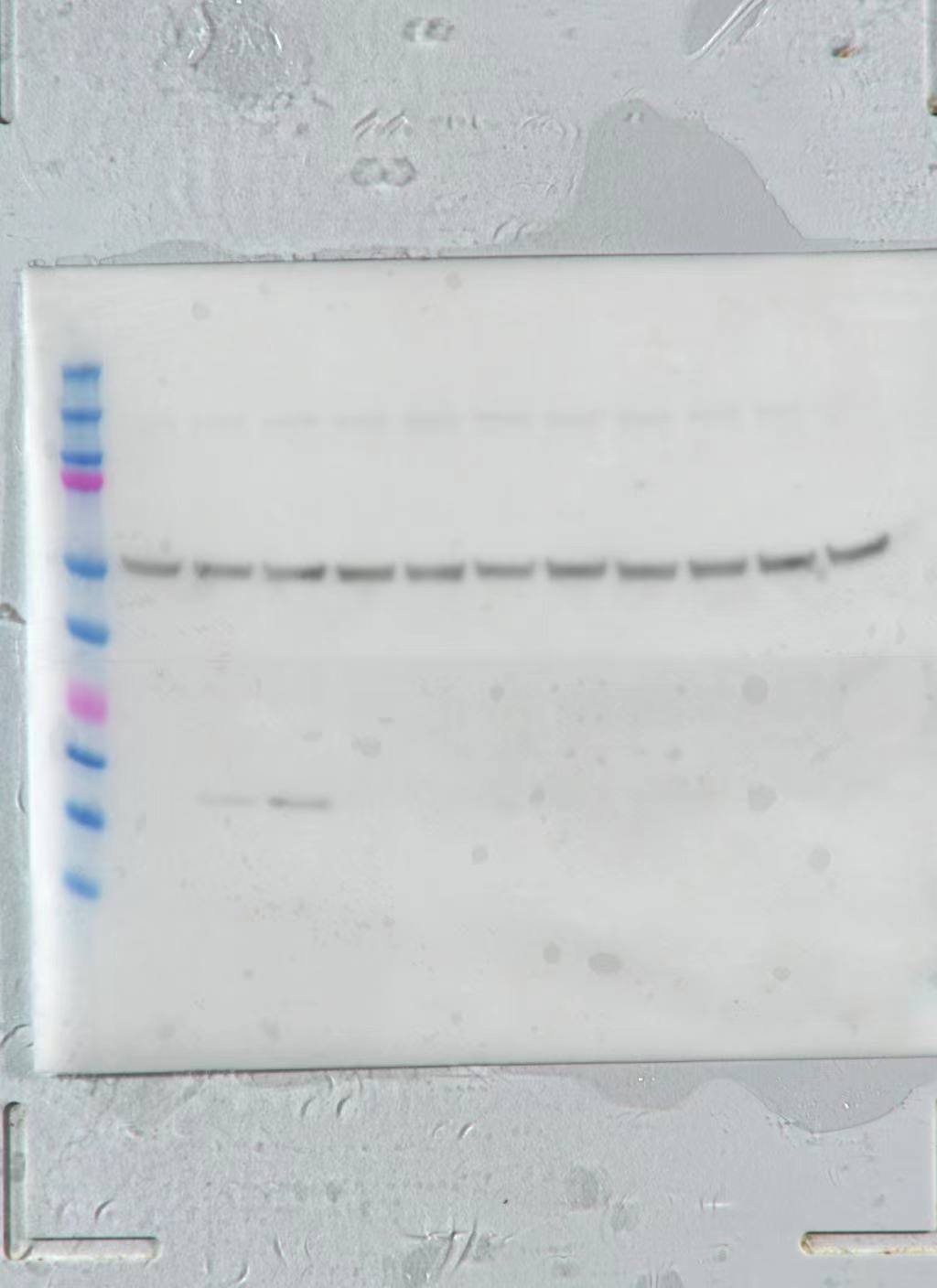

Supplement: Supplementary file 6 — Source Data [file 41467_2023_39151_MOESM6_ESM.zip › source data/Fig_S3c_blots/Tub_with marker.jpg]

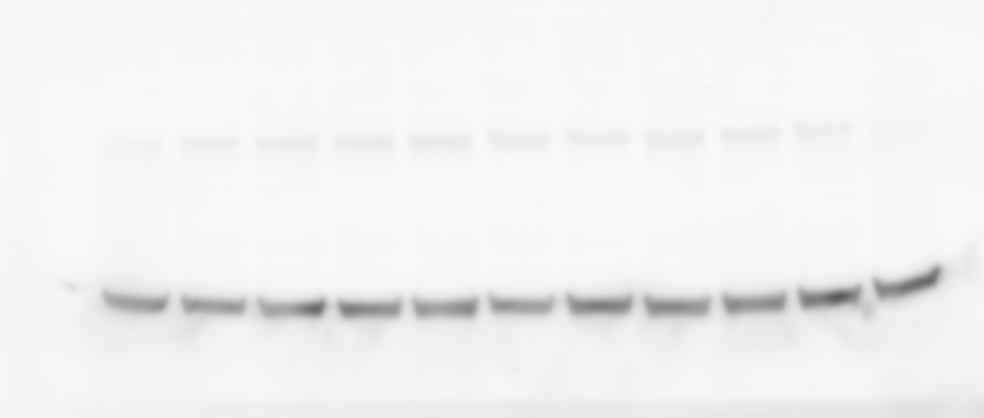

Supplement: Supplementary file 6 — Source Data [file 41467_2023_39151_MOESM6_ESM.zip › source data/Fig_S3c_blots/Tubulin all.tif]

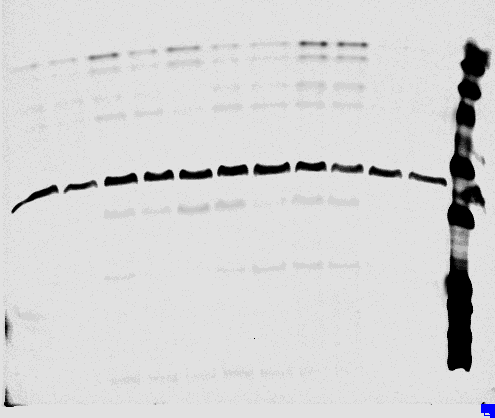

Supplement: Supplementary file 6 — Source Data [file 41467_2023_39151_MOESM6_ESM.zip › source data/Fig_S3e_blots/anti-global AcK_Actin_bw.png]

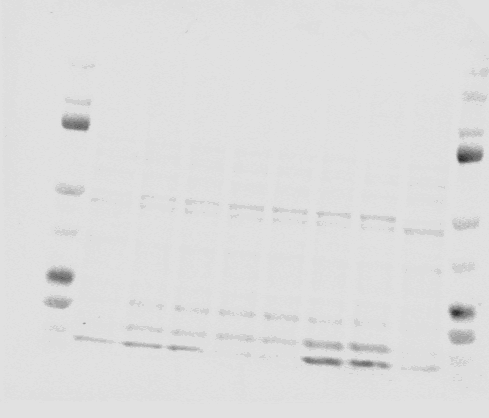

Supplement: Supplementary file 6 — Source Data [file 41467_2023_39151_MOESM6_ESM.zip › source data/Fig_S3f_blots/LA_td treatment_HeLa013_AcK_bw.png]

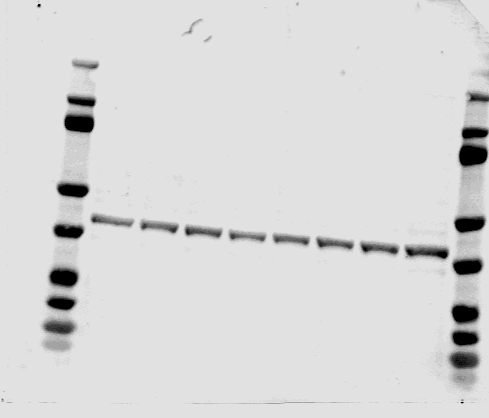

Supplement: Supplementary file 6 — Source Data [file 41467_2023_39151_MOESM6_ESM.zip › source data/Fig_S3f_blots/LA_td treatment_HeLa013_actin_bw.png]

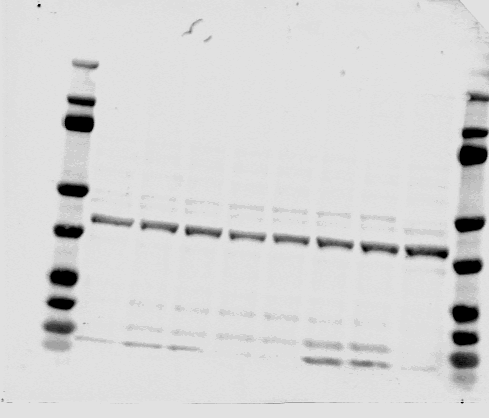

Supplement: Supplementary file 6 — Source Data [file 41467_2023_39151_MOESM6_ESM.zip › source data/Fig_S3f_blots/LA_td treatment_HeLa013_combined_bw.png]
