# Supplementary material for: Chemoproteomic target deconvolution reveals Histone Deacetylases as targets of (R)-lipoic acid
Source: Nat Commun. 2023 Jun 15;14:3548. doi: 10.1038/s41467-023-39151-8 (PMC10272112; doi:10.1038/s41467-023-39151-8)
Supplement: Supplementary file 2 — Peer Review File [file 41467_2023_39151_MOESM2_ESM.pdf]

nature portfolio

Peer Review File

Chemoproteomic target deconvolution reveals Histone Deacetylases as targets of (R)-lipoic acid

Open Access This file is licensed under a Creative Commons Attribution 4.0 International License, which permits use, sharing, adaptation, distribution and reproduction in any medium or format, as long as you give appropriate credit to the original author(s) and the source, provide a link to the Creative Commons license, and indicate if changes were made. In the cases where the authors are anonymous, such as is the case for the reports of anonymous peer reviewers, author attribution should be to 'Anonymous Referee' followed by a clear attribution to the source work. The images or other third party material in this file are included in the article's Creative Commons license, unless indicated otherwise in a credit line to the material. If material is not included in the article's Creative Commons license and your intended use is not permitted by statutory regulation or exceeds the permitted use, you will need to obtain permission directly from the copyright holder. To view a copy of this license, visit http://creativecommons.org/licenses/by/4.0/.

REVIEWER COMMENTS

Reviewer #1 (Remarks to the Author):

Lipoic acid (LA) is a disulfide-containing fatty acid, and as such it is very likely that its mechanism of action involves redox reactions. Here the authors performed chemoproteomics-aided target deconvolution of LA and its active analogue lipoamide. They found that histone deacetylases are molecular targets of the reduced form of both LA and lipoamide. They also found that only R-enantiomer inhibits HDACs at physiologically relevant concentrations.

Indeed, LA exists in nature as R enantiomer, while synthetic supplementation consists of a racemic composition of R and S forms. The belief that the R enantiomer of LA is the one responsible for biological activity is widely spread in literature. For instance, only the R enantiomer of LA function as a cofactor of respiratory chain enzymes (e.g. Arch Biochem Biophys. 1989 Feb 1;268(2):465-74).

The finding that lipoid acid inhibits HDACs is not altogether novel either. Myzak et al (Mol Carcinog. 2006 Jun; 45(6): 443–446) speculated that lipoic acid might be an HDAC inhibitor, and Dashwood and Ho asserted that in several publications (e.g., PMC2737738). The fact that these sources are not cited reduces the scholarly level of this study.

The chemoproteomics method used to discover LA targets are not novel, and no details are provided (e.g., the total number of proteins analysed, the scoring system, the score distribution, etc).

The choice of A549 cells for testing the stress granule formation is not justified. The ROS analysis didn't seem to be performed, even though the main effect of HDAC inhibition should be redox-related.

Summarizing, while HDAC inhibition is very likely one of the LA effects in cells, the authors failed to demonstrate that this inhibition accounts for the bulk of biological effects of LA.

Reviewer #2 (Remarks to the Author):

The manuscripts entitled: "Chemoproteomic target deconvolution of lipoid acid reveals histone deacetylases as the main targets", submitted by Kuster and coworkers investigates the interaction of lipoic acid stereoisomers and lipoamide with HDAC enzymes in cell lysate, against recombinant enzymes, as well as effects on lysine acetylation in cells. Finally, the effect of compounds on granule formation is investigated in A549 cancer cells, indicating the mechanism of action to be via HDAC inhibition rather than intracellular redox chemistry-related.

The manuscript is very well written and illustrated, and the discoveries are of significant interest to the scientific community.

I only recommend a couple of very small changes:

(1) Relating to discussion of Fig 2d at the bottom of page 4, where it is stated that the HDAC inhibition profile is similar to that of SAHA and ricolinostat, which is not obvious from the data, since the drugs are much more potent and selectivity profiles appear to differ somewhat as well.

(2) The discussion on page 5 of the effects of compound treatment on cellular lysine acetylation. Here the authors state that such experiments demonstrate target engagement, which is not technically correct. Indirectly, changes in tubulin acetylation only indicate HDAC6 inhibition in the cells.

Technical comments:

In figure 3, it is not clear how many replicates were performed for panel a, and statistics should be applied for the data in all panels. Usually, biological triplicates are recommended for western blot analyses.

The full blots should also be shown as supporting information.

Reviewer #3 (Remarks to the Author):

Lipoic acid (LA) is an interesting disulfide-containing fatty acid with diverse biological activities and therapeutic potentials. As an endogenous metabolite, LA acts as an essential enzyme cofactor in central metabolic pathways via lipoylation, but the latter seems not to be a widely spread PTM. As a widely used food supplement/pharmaceutical with extraordinarily high doses, the mode of action of LA is historically attributed to its metal ion chelation, antioxidant and ROS scavenging properties, owing to the dithiolane ring. Intriguingly, Lechner et al report an unprecedented MoA of LA and its analog lipoamide (LM), which is uncovered by the state-of-the-art chemoproteomic platform. That is, the reduced form of LA and LM binds and inhibits a group of HDACs, leading to the hyperacetylation of their enzymatic substrates and likely the regulation of stress granule formation.

Overall, this work is conceptually novel and technically sound from the proteomic point of view. In addition, the manuscript is well-written and inspiring to read. However, my enthusiasm to this work is dampened by the following crucial issues.

1. The Kd/EC50 values of (R/S)-LA and (R/S)-LM on HDACs are generally in the µM range, while the plasma concentration of LA is suggested to be in the mM range. It apparently raises the question that why it is required to use extraordinarily high doses of LA to achieve beneficial effects in clinical trials. In other words, whether the biological effects of LA can be mainly attributed to the binding and inhibition of HDACs.

2. Clearly, only the reduced form of LA and LM can bind and inhibit HDACs, which represents the most important premise of the proposed MoA. Although it has been previously suggested that cyclic 5-member disulfides can be reduced non-specifically in biological systems (Felber et al., Nat Commun, 2022), it is not known whether LA and LM are fully reduced in cells or what's the percentage of their reduced forms.

3. Indeed, (R/S)-LA and (R/S)-LM led to increase of acetylation of the selected HDAC substrates at concentrations as low as 50 µM and in a concentration-dependent fashion. However, the boost of acetylation signals was mainly induced by those chemicals in the mM range. Can such an effect be also attributed by the redox regulation of HDACs upon the treatment of those compounds? This concern might be easily addressed by including (S)-LA as a negative control. Regardless, one cannot to rule out the possibility that the increase of acetylation may also be caused by a HDAC-independent pathway, which should be at least discussed.

4. It is very interesting to observe the stereoselective effects of LA against HDACs; (S)-LA shows no activity. Unfortunately, the reviewer failed to find any attempt to explain this. The authors are therefore suggested to experimentally measure the binding kinetics of (R)-LA and (S)-LA and/or theoretically calculate the reducing potential values of these enantiomeric chemicals. Also, the authors are suggested to test enantiomeric LMs if available commercially; will (S)-LM also show no activity against any HDAC?

Minor:

Fig. 1c, 'LM' is lost under the x-axis.

The authors claimed that "1 mM lipoic acid would increase ROS scavenging thiol by only 2-4%", while this is based upon a rough estimation of the intracellular concentration of free thiols.

Point-by-point response

The text provided by the the reviewers was kept as is (in black) and our responses are given in blue italic font.

Reviewer #1 (Remarks to the Author):

Lipoic acid (LA) is a disulfide-containing fatty acid, and as such it is very likely that its mechanism of action involves redox reactions. Here the authors performed chemoproteomics-aided target deconvolution of LA and its active analogue lipoamide. They found that histone deacetylases are molecular targets of the reduced form of both LA and lipoamide. They also found that only R-enantiomer inhibits HDACs at physiologically relevant concentrations.

Indeed, LA exists in nature as R enantiomer, while synthetic supplementation consists of a racemic composition of R and S forms. The belief that the R enantiomer of LA is the one responsible for biological activity is widely spread in literature. For instance, only the R enantiomer of LA function as a cofactor of respiratory chain enzymes (e.g. Arch Biochem Biophys. 1989 Feb 1;268(2):465-74).

The finding that lipoid acid inhibits HDACs is not altogether novel either. Myzak et al (Mol Carcinog. 2006 Jun; 45(6): 443–446) speculated that lipoic acid might be an HDAC inhibitor, and Dashwood and Ho asserted that in several publications (e.g., PMC2737738). The fact that these sources are not cited reduces the scholarly level of this study.

The authors are indeed aware of the publications by Myzak and Dashwood (and now cite this prior work). In their first review, (Mol Carcinog. 2006; DOI 10.1002/mc.20224) they speculate that Lipoic acid might bind to HDACs via its carboxylic acid moiety. They propose an HDAC binding and inhibition mode similar to the well-known dietary compound butyric acid and suggest that dietary molecules such as Biotin or garlic-derived compounds also bind and inhibit HDACs in a similar way. No experimental evidence is provided for this speculation. In their second review (the one mentioned by this reviewer, Dashwood and Ho, 2007, doi:10.1016/j.semcancer.2007.04.001), they repeat this proposal without (i) referencing any study that would have shown this or (ii) providing own data supporting this hypothesis. Indeed, carboxylic acids such as butyrate can inhibit HDACs. We and others (Bantscheff et al., Nat Biotech, 2011. Lechner et al., Nat Chem Bio, 2023) have shown that this is true for Valproic acid and Phenylbutyric acid. However, binding affinities of these carboxylic acids are 10 to 100-fold less potent (~300-500 µM EC50s) than the ones observed for Lipoic acid (5-30 µM EC50s) in comparable chemoproteomic competition assays. In contrast to Mysak and Dashwood, we suggest that the binding of Lipoic acid is mediated via one or two of its reduced thiol groups, similar to well-established HDAC inhibitors Romidepsin and Psammaplin. We are confident that this is the true binding mechanism because:

a) Only reduced Lipoic acid/Lipoamide but not the oxidized forms bind HDACs (a chemical change at the carboxy-distal part of the molecule)
b) Lipoamide binds and inhibits HDACs with an affinity similar to the one of Lipoic acid. Importantly, Lipoamide does not have a carboxy group that is suggested by Mysak et al. to be the zinc-chelating warhead relevant for HDAC inhibition.
c) More important still, the affinity matrix employed for the initial target identification experiment links lipoic acid to sepharose beads via an amidation reaction of the carboxylic acid. If the carboxylic acid was relevant for HDAC target binding, amidation and addition of the sterically demanding linker would completely abolish HDAC binding and the ability to pull

it down with the affinity matrix. Based on our experiments, this was clearly not the case, demonstrating that lipoic acid does not bind to HDACs via its carboxylic acid.

Still, we now mention the HDAC inhibition mechanism proposed by Myzak, Dashwood et al. in our discussion:

> *"Importantly, the same argument clearly opposes the published hypothesis that lipoic acid might inhibit HDACs akin to other nutritional short chain fatty acids by zinc chelation via its carboxy group[26, 27]."*
>
> *26. Myzak, M.C., Ho, E. & Dashwood, R.H. Dietary agents as histone deacetylase inhibitors. Mol Carcinog 45, 443-446 (2006).*
>
> *27. Dashwood, R.H. & Ho, E. Dietary histone deacetylase inhibitors: from cells to mice to man. Semin Cancer Biol 17, 363-369 (2007).*

The chemoproteomics method used to discover LA targets are not novel, and no details are provided (e.g., the total number of proteins analysed, the scoring system, the score distribution, etc).

Correct. The method is in fact well established now and proved useful for the purpose of the current study. All the requested information (and much more) can be found in the files deposited with the PRIDE repository. To inform readers interested in these numbers, we changed the text:

> *"Amongst the three cell lines tested, HDACs were the only confidently identified targets across the 1500-3000 proteins quantified in these assays"*

We did not declare confident targets by a scoring system but following published procedures that we now cite in the manuscript. We also extended the description of the process for identifying targets in the methods section:

> *"Targets of the inhibitors were annotated manually according to published procedures[12, 46, 47]. In brief, a protein was considered a target or interactor of a target if the resulting binding curve showed a sigmoidal curve shape with a dose-dependent decrease of binding to the beads. Additionally, the number of unique peptides and MSMS counts per condition were taken into account. Positive target binding across several independent experiments performed with different cell lysates further substantiated our confidence for a true positive drug-target binding event."*
>
> *12. Lechner, S. et al. Target deconvolution of HDAC pharmacopoeia reveals MBLAC2 as common off-target. Nat Chem Biol 18, 812-820 (2022).*
>
> *46. Klaeger, S. et al. The target landscape of clinical kinase drugs. Science 358 (2017).*
>
> *47. Reinecke, M. et al. Chemoproteomic Selectivity Profiling of PIKK and PI3K Kinase Inhibitors. ACS Chem Biol 14, 655-664 (2019).*

We now also provide supplementary .pdf files via the MassIVE proteomics database. The .pdf files show protein intensity-based as well as MS/MS count- and peptide ID-based dose-response curves for each protein identified via LC-MS/MS. These files were used for initial data analysis and should simplify the data assessment by reviewers and readers.

For reviewer access please follow the link

https://massive.ucsd.edu/ProteoSAFe/dataset.jsp?task=89a720aaa25642eea8ff2fdb0926e1f9

and sign in with

User name: MSV000091758_reviewer

Password: lipoic_acid

Data is easily accessible for download by clicking the "Browse Dataset Files" button on the right

The choice of A549 cells for testing the stress granule formation is not justified. The ROS analysis didn't seem to be performed, even though the main effect of HDAC inhibition should be redox-related.

Choice of cell line: A549 cells were chosen because these had previously shown to be suitable for measuring stress granule formation including yielding high-quality microscopic pictures required for the stress granule assay. We also performed additional chemoproteomic experiments in this cell line showing that HDACs (and their complex partners) are the only confidently identified lipoic acid target proteins in A549 cells (see figure below).

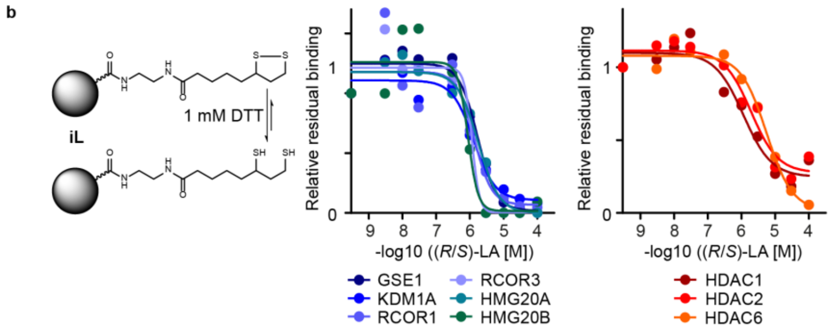

***"Supplementary Fig. 1|Chemoproteomics identifies HDACs as targets of lipoic acid and lipoamide.** […]. **b**, Dose-response curves of HDACs and CoREST complex partners from the LA-iL competition experiment in A549 cell lysate."*

*ROS analysis: For the original arsenite-induced stress granule experiments, we used an HDAC inhibitor as a positive control. It has a comparable effect to R-LA. We included S-LA as a negative control to show that this is not a redox effect (as the redox potential of R-LA and S-LA should be identical). We believe that this is indeed a very strong control (possibly the best possible because the chemical composition of the R-LA and S-LA is identical). Still, we now performed ROS analysis under the same assay conditions as used for the stress granule experiment. The data clearly shows that arsenite is not inducing significant ROS and that lipoic acid treatment does not significantly alter ROS levels under these conditions (see figure below). While both S-LA and R-LA show comparable ROS-buffering effects under extreme conditions (200 uM tBuOOH) (**Fig. 4c**), only R-LA has effects on HDAC substrate hyperacetylation (**Fig. 3b, Supplementary Fig. 3c-d**), intracellular HDAC target engagement (**Fig. 3d**), and stress granule formation (**Fig. 4a-b**). We can therefore clearly separate the observed HDAC-linked (and R-LA-specific) phenotypes from (common) redox-related effects.*

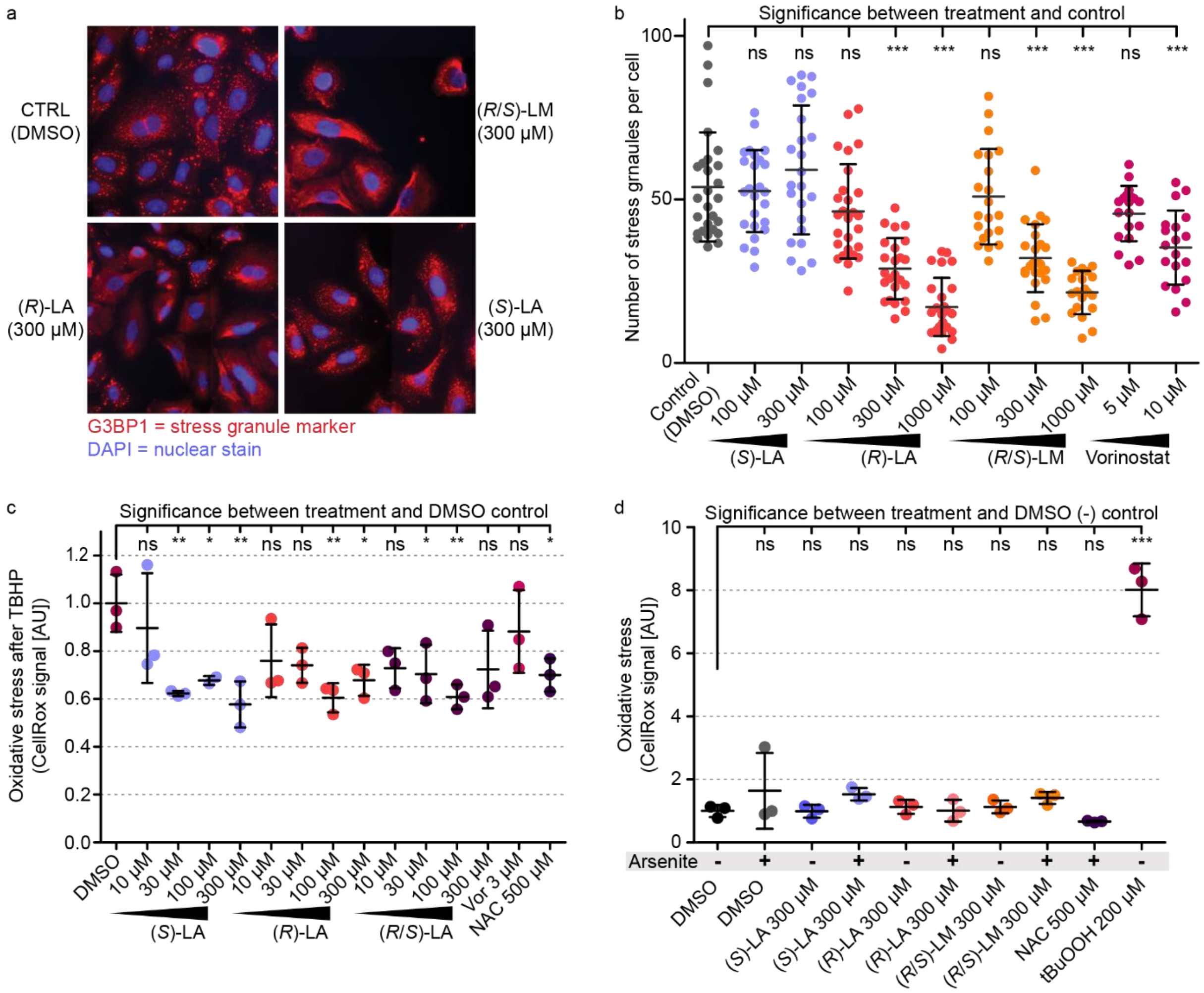

Figure 4 | Lipoic acid and lipoamide reduce stress granule formation in cells. a, Immunofluorescence detection of the stress granule marker G3BP1 in A549 cancer cells. Stress granules appear as red foci in the DMSO control and cells treated with (HDAC-inactive) (*S*)-LA. The reduction of defined stress granules in response to (*R*)-LA and (*R*/*S*)-LA is apparent from the blurred red areas. **b,** Quantification of the number of stress granules per cell. Each treatment was performed in n = 3 independent biological experiments and between 140 and 150 cells were submitted to stress granule counting. **c,** Levels of oxidative stress induced by 2 h treatment with 200 µM Tertbutylhydroperoxide (BuOOH) after 1h pre-treatment with drugs (Vor: Vorinostat, NAC: N-acetylcysteine) in A549 cells. Oxidative stress levels were assessed with the CellRox assay. Every data point corresponds to one biological replicate and is the mean CellRox intensity from 9-10 pictures capturing 60 – 180 cells in total. **d,** Levels of oxidative stress in A549 cells after 1.5 h drug pre-treatment, optionally followed by a 30 min arsenite pulse. Oxidative stress levels were assessed with the CellRox assay. Every data point corresponds to one biological replicate and equals the mean CellRox intensity from 10 - 15 pictures capturing 60 – 180 cells in total. **b – d**, The significance was calculated between the control and drug pre-treatments by one-way ANOVA following the Dunnett test for multiple comparison via the GraphPad Prism software. Data are presented as means ± SD. ns: not significant; *** : P-value ≤ 0.001, ** : P-value ≤ 0.01, * : P-value ≤ 0.05 in one-way ANOVA after Dunnett's multiple comparison test).

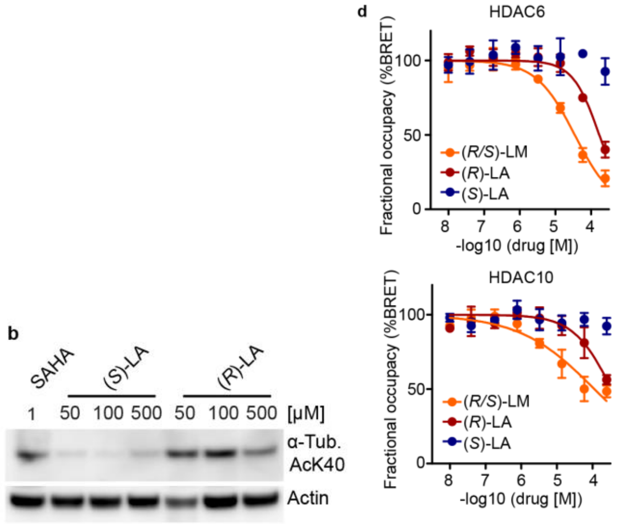

Figure 3| *[…]* ***b,*** *Western blot for α-Tubulin AcK40 acetylation levels after 12 h treatment of A549 cells with SAHA (Vorinostat), (R)-LA, and (S)-LA. […]* ***d,*** *HDAC6 and HDAC10 nano-BRET assays for in-cellulo target engagement in HEK293T cells (n = 3 independent experiments, curve fitted with a variable slope; bottom constrained to 0 and top constrained to 100).*

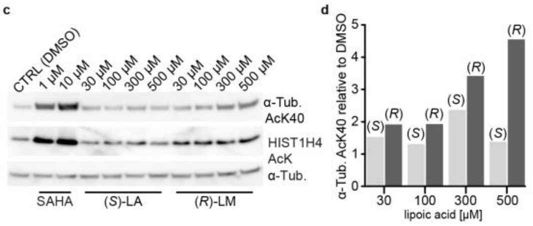

Supplementary Fig. 3| *[…]* ***c,*** *Western blot of HEK293T cells treated for 6 h with SAHA (Vorinostat), (R)-lipoic acid, or (S)-lipoic acid.* ***d,*** *comparison of dose-dependent effect of R and S-LA treatment on alpha-Tub AcK in HEK293 cells (quantification data from Supplementary Fig. 3c)*

Summarizing, while HDAC inhibition is very likely one of the LA effects in cells, the authors failed to demonstrate that this inhibition accounts for the bulk of biological effects of LA.

Please also see our responses above. The authors agree that we cannot exclude the possibilities that there are more targets of LA that could account for its cellular effects. However, to summarize why we think that HDAC inhibition is an important MoA: Both the potency of target binding (Kd of 5-30 uM) and the potency of intracellular target engagement as well as HDAC substrate hyperacetylation in the range of 50-300 uM are well in line with the 500 uM peak plasma concentration of LA that can be reached in patients. We envision that, in the future, using S-LA and R-LA as chemical probe pair will allow scientists to study the molecular basis for further phenotypic effects of lipoic acid that may or may not be mediated by HDAC inhibition, redox biology or metal binding. Because we cannot exclude other MoAs, we cautiously step back from claiming HDACs to be the 'main targets' by changing the title as follows:

> *"Chemoproteomic target deconvolution reveals Histone Deacetylases as targets of (R)-lipoic acid"*

We have also reduced the strength in the wording throughout the text and mention that there may be other targets that drive certain effects:

> *"In line with this idea, chemoproteomic affinity profiling using immobilized lipoic acid identified Zn^{2+}-dependent HDACs as the only proteins bound specifically by lipoic acid and lipoamide. This does not exclude the possibility that other targets may exist, as pulldown experiments were only performed in MV4-11, SW620, and A549 cells, which may not express all potential target proteins. Of note, the affinity matrix was created by immobilizing (R/S)-LA via an amidation of its carboxylic acid group. Any target protein that may rely on an interaction with the negatively charged carboxy-group, would not score in the assay."*

Reviewer #2 (Remarks to the Author):

The manuscripts entitled: "Chemoproteomic target deconvolution of lipoid acid reveals histone deacetylases as the main targets", submitted by Kuster and coworkers investigates the interaction of lipoic acid stereoisomers and lipoamide with HDAC enzymes in cell lysate, against recombinant enzymes, as well as effects on lysine acetylation in cells. Finally, the effect of compounds on granule formation is investigated in A549 cancer cells, indicating the mechanism of action to be via HDAC inhibition rather than intracellular redox chemistry-related.

The manuscript is very well written and illustrated, and the discoveries are of significant interest to the scientific community.

Thank you.

I only recommend a couple of very small changes:

(1) Relating to discussion of Fig 2d at the bottom of page 4, where it is stated that the HDAC inhibition profile is similar to that of SAHA and ricolinostat, which is not obvious from the data since the drugs are much more potent and selectivity profiles appear to differ somewhat as well.

We changed the text to clarify our statement:

> *"In summary, the above data indicate that reduced (R)-LA, as well as (R/S)-LM feature an HDAC selectivity profile similar (albeit less potent) compared to that of clinical drugs such as Vorinostat and Ricolinostat (Fig. 2d, Supplementary Fig. 2f). All these molecules inhibit HDAC1-3 with similar affinity and HDAC6 with 5 to 15-fold higher relative potency."*

(2) The discussion on page 5 of the effects of compound treatment on cellular lysine acetylation. Here the authors state that such experiments demonstrate target engagement, which is not technically correct. Indirectly, changes in tubulin acetylation only indicate HDAC6 inhibition in the cells.

*Fair point. So far, combining in vitro data for HDAC binding and inhibition as well as substrate hyperacetylation has been the "gold standard" to demonstrate HDAC inhibition in cells. Using now S-LA as a negative control (see new experiments) provides additional confidence that the observed acetylation effects are not indirectly mediated by lipoic acid redox or metal ion chelating properties (see above, **Fig. 3b, Supplementary Fig. 3. c-d**). To answer the question directly, we performed new nanoBRET experiments demonstrating HDAC6 and HDAC10 binding in cells. These results are in line with the western blot data (see above, **Fig. 3d**), such that R-LA binds to HDAC6/10 in cells, while S-LA does not bind to HDAC6/10 at assayed concentrations.*

Technical comments:

In figure 3, it is not clear how many replicates were performed for panel a, and statistics should be applied for the data in all panels. Usually, biological triplicates are recommended for western blot analyses.
The full blots should also be shown as supporting information.

The experiment underlying Fig. 3a was actually not carried out in replicates. Instead, we performed a dose-dependent analysis which are statistically even more powerful than replicates using single concentrations. Statistical tests were applied wherever replicates were measured. We now provide the full blots as part of the source data with the paper.

Reviewer #3 (Remarks to the Author):

Lipoic acid (LA) is an interesting disulfide-containing fatty acid with diverse biological activities and therapeutic potentials. As an endogenous metabolite, LA acts as an essential enzyme cofactor in central metabolic pathways via lipoylation, but the latter seems not to be a widely spread PTM. As a widely used food supplement/pharmaceutical with extraordinarily high doses, the mode of action of LA is historically attributed to its metal ion chelation, antioxidant and ROS scavenging properties, owing to the dithiolane ring. Intriguingly, Lechner et al report an unprecedented MoA of LA and its analog lipoamide (LM), which is uncovered by the state-of-the-art chemoproteomic platform. That is, the reduced form of LA and LM binds and inhibits a group of HDACs, leading to the hyperacetylation of their enzymatic substrates and likely the regulation of stress granule formation. Overall, this work is conceptually novel and technically sound from the proteomic point of view. In addition, the manuscript is well-written and inspiring to read.

However, my enthusiasm to this work is dampened by the following crucial issues.

1. The Kd/EC50 values of (R/S)-LA and (R/S)-LM on HDACs are generally in the µM range, while the plasma concentration of LA is suggested to be in the mM range. It apparently raises the question that why it is required to use extraordinarily high doses of LA to achieve beneficial effects in clinical trials. In other words, whether the biological effects of LA can be mainly attributed to the binding and inhibition of HDACs.

*Our read of the literature is that this perceived discrepancy does not exist. The peak plasma concentration of LA is about 500 uM (see ref. [10]), which is only ~30 times higher than the Kd for HDAC binding in pulldown assays and only 2-3 times higher than target engagement (nanoBRET) and substrate hyperacetylation in cells (**Fig. 3b, Supplementary Fig. 3a, c-d, Fig. 3d**). Hence, a plasma concentration of LA in the range of 500 uM would actually be required to achieve a meaningful level of HDAC inhibition in cells.*

10. Dorsam, B. & Fahrer, J. The disulfide compound alpha-lipoic acid and its derivatives: A novel class of anticancer agents targeting mitochondria. Cancer Lett 371, 12-19 (2016).

2. Clearly, only the reduced form of LA and LM can bind and inhibit HDACs, which represents the most important premise of the proposed MoA. Although it has been previously suggested that cyclic 5-member disulfides can be reduced non-specifically in biological systems (Felber et al., Nat Commun, 2022), it is not known whether LA and LM are fully reduced in cells or what's the percentage of their reduced forms.

Felber et al. (Nat Comm, 2022) confirmed that the strained dithiolane ring of LA/LM is readily reduced by thiol-containing molecules without the need for enzymatic assistance. Our in vitro data shows that

only the reduced forms can inhibit HDACs and our western blot as well as nanoBRET assays provide evidence for direct in cellulo target engagement and inhibition. We therefore assume that a substantial fraction of LA is reduced intracellularly. However, we agree with the reviewer that the actual percentage of intracellular LA available in reduced form is not known. Indeed, an incomplete reduction might be one of the reasons, why around 10 to 100-fold higher levels of LA are needed to achieve complete HDAC inhibition in cellulo compared to in vitro assays. We now mention this consideration in our discussion:

> *"Cellular HDAC inhibition by lipoic acid occurred at 10 to 100-fold higher concentrations compared to the in vitro recombinant HDAC inhibition assay. This might be explained by incomplete intracellular reduction, lower intracellular compound concentration, or metabolic conversion of lipoic acid."*

3. Indeed, (R/S)-LA and (R/S)-LM led to increase of acetylation of the selected HDAC substrates at concentrations as low as 50 µM and in a concentration-dependent fashion. However, the boost of acetylation signals was mainly induced by those chemicals in the mM range.

This is correct. See also our response above. Our western blot and nano-BRET results suggest that HDACs are inhibited intracellularly with EC50s in the range of 100-300 uM. This means that complete HDAC inhibition might be achieved only at concentrations 3-5x EC50 (i.e. as high as 0.3 – 1.5 mM. Of note, even partial HDAC inhibition may already induce clear phenotypes and might actually be desirable for therapeutic purposes, because complete, persistent, and systemic HDAC inhibition would lead to toxic side effects in patients (a recurrent issue of synthetic potent HDAC inhibitors).

Can such an effect be also attributed by the redox regulation of HDACs upon the treatment of those compounds? This concern might be easily addressed by including (S)-LA as a negative control. Regardless, one cannot to rule out the possibility that the increase of acetylation may also be caused by a HDAC-independent pathway, which should be at least discussed.

*Please also see our responses above. We performed additional experiments using pure forms of S-LA and R-LA and indeed observed hyperacetylation of HDAC substrates only for R-LA treatment (**Fig. 3b, Supplementary Fig. 3c-d**). Moreover, additional nanoBRET experiments demonstrated direct in-cell HDAC target engagement for R-LA (**Fig. 3d**). Additional experiments showed that lipoic acid did not significantly influence ROS levels under standard cell culture conditions. Together, these findings suggest that ROS/redox effects are not related to HDAC substrate hyperacetylation.*

4. It is very interesting to observe the stereoselective effects of LA against HDACs; (S)-LA shows no activity. Unfortunately, the reviewer failed to find any attempt to explain this. The authors are therefore suggested to experimentally measure the binding kinetics of (R)-LA and (S)-LA and/or theoretically calculate the reducing potential values of these enantiomeric chemicals. Also, the authors are suggested to test enantiomeric LMs if available commercially; will (S)-LM also show no activity against any HDAC?

We agree that the stereoselectivity is highly interesting in principle but the authors defer such investigations to future studies for a number of reasons. First, enantiomeric pure forms of LM are not commercially available. Second, because S-LA does not bind HDACs at all, we cannot perform experiments to compare R-LA and S-LA binding kinetics. Third, we tried to determine the crystal structure of R-LA bound to HDACs to better understand the binding mode. Unfortunately, and despite multiple attempts, we failed to obtain co-crystals. Still, some considerations regarding the possible binding mode are plausible. The stereocenter is located next to one thiol group and, therefore, is buried

in the narrow active site of HDACs. We hypothesize that the stereocenter-thiol group of reduced S-LA would sterically clash with active site amino acid residues or significantly increase binding energy. Therefore, S-LA either cannot enter the active site at all or suffers a reorientation of the thiol groups in a way that prevents proper Zn-chelation.

In theory, the reducing potential and ROS scavenging capability should not depend on the stereochemistry. As expected, our new ROS experiments showed that there is no difference between S-LA and R-LA in their ability to buffer ROS under high peroxide conditions.

Minor:
Fig. 1c, 'LM' is lost under the x-axis.

We have fixed this issue.

The authors claimed that "1 mM lipoic acid would increase ROS scavenging thiol by only 2-4%", while this is based upon a rough estimation of the intracellular concentration of free thiols.

Yes, this was a rough but likely conservative estimation considering that methionine and other biomolecules can also act as "ROS scavengers" and were not included in our estimation. Further support for the notion that ROS scavenging by lipoic acid is a lesser or even neglectable effect comes from several reviews by oxidative stress experts that clearly state that the scavenging of ROS by small molecules plays an irrelevant role compared to the ROS consumption by enzymes.

22. Forman, H.J. & Zhang, H. Targeting oxidative stress in disease: promise and limitations of antioxidant therapy. Nat Rev Drug Discov 20, 689-709 (2021).

23. Murphy, M.P. et al. Guidelines for measuring reactive oxygen species and oxidative damage in cells and in vivo. Nat Metab 4, 651-662 (2022).

24. Sies, H. et al. Defining roles of specific reactive oxygen species (ROS) in cell biology and physiology. Nat Rev Mol Cell Biol 23, 499-515 (2022).

Interestingly, while these reviews summarize the effects of well-established antioxidant molecules, lipoic acid is not mentioned even once in this context further adding to the notion that the role of lipoic acid as an antioxidant is not clearly worked out yet.

REVIEWERS' COMMENTS

Reviewer #1 (Remarks to the Author):

Am satisfied with authors' response

Reviewer #3 (Remarks to the Author):

The authors addressed all my concerns. Therefore, I support its publication as it is in Nat Commun.
